# Mapping lncRNAs onto multilevel mRNA co‑expression modules in autism

**DOI:** 10.1038/s41398-026-04275-0

**Published:** 2026-07-13

**Authors:** Chen-Ling Lee, Geng-Ming Hu, Yi-Pei Li, Te-Lun Mai

**Affiliations:** 1 Department of Life Science, National Taiwan University, Taipei, Taiwan; 2 Department of Physics, National Taiwan Normal University, Taipei, Taiwan; 3 Center for Computational and Systems Biology, National Taiwan University, Taipei, Taiwan

**Keywords:** Personalized medicine, Autism spectrum disorders

## Abstract

Autism spectrum disorder (ASD) involves heterogeneous genetic and transcriptomic alterations, but how these changes are organized across hierarchical co-expression scales and linked to long noncoding RNAs (lncRNAs) remains incompletely understood. Here, we present Minimum Span Clustering Network (MSCN), an unsupervised, deterministic framework that constructs traceable multilevel mRNA co-expression hierarchies without requiring soft-thresholding powers, fixed module numbers, cut heights, or stochastic initialization. Applied to two independent ASD brain transcriptome cohorts, MSCN reveals hierarchical gene modules across four resolution levels, enabling the detection of transcriptional patterns ranging from low-level, specific signals to high-level, broader biological pathways. We uncovered modules enriched in neuronal/axonal, developmental, and immune-related pathways, reflecting interconnected neurodevelopmental and immune-dysregulation programs in ASD. Cross-method comparisons showed that MSCN complements WGCNA and MEGENA by preserving biologically concordant modules while providing an explicit parent-child hierarchy; simulation and benchmark analyses supported comparable module recovery, preservation, and enrichment performance. By mapping lncRNAs to MSCN-derived mRNA modules, we identified lncRNAs associated with ASD-related mRNA modules and evaluated them as candidate statistical mediators in downstream transcription factor (TF)-lncRNA-mRNA analyses. This analysis identified over 11,000 candidate TF-lncRNA-mRNA axes, including 644 axes involving 46 SFARI score 1 or 2 genes, four of which were syndromic genes, and 12 named lncRNA mediators. External transcriptomic evaluation further supported 789 axes, including representative *HIF1A-STXBP5-AS1-CADPS*, *E2F1-PART1-SCN2A*, and *RELA-RFPL1S-GRIN2A* relationships. Together, these findings establish MSCN as a scalable framework for decomposing ASD mRNA co-expression architecture, provide a hypothesis-generating resource linking coding and noncoding transcriptomic alterations, and help prioritize these relationships for future validation.

## Introduction

Autism spectrum disorder (ASD) is a complex neurodevelopmental condition marked by extensive genetic heterogeneity and polygenic risk [1–3]. While genome-wide association studies and exome sequencing have identified hundreds of risk genes, the effect of each associated variant is modest, and their collective impact on ASD pathophysiology remains poorly understood [4–7]. Increasing evidence suggests that these risk variants converge upon a limited set of molecular pathways, particularly those regulating synaptic function, neuronal development, and immune responses [8–10]. Transcriptomic analyses of postmortem ASD brain further support this view, revealing recurrent cross-cohort molecular signatures. A seminal co-expression study identified ASD-associated neuronal and immune-glial modules and attenuated frontal-temporal regional patterning [11]; subsequent large-scale analyses showed widespread gene- and isoform-level dysregulation, including neuronal/synaptic down-regulation and astrocytic, microglial, and interferon-related immune up-regulation [12]. Together, these findings show convergent ASD transcriptomic signatures across cohorts, underscoring the need to resolve their hierarchical organization. Transcriptomic network modeling, which infers putative functional relationships from shared transcriptional patterns, has therefore emerged as a promising strategy for addressing this challenge [13–15]. Prominent methods, such as Weighted Gene Co-expression Network Analysis (WGCNA), have been used to identify disease-associated modules in psychiatric and neurodevelopmental disorders, including autism, schizophrenia, bipolar disorder, and epilepsy [16–19]. Nevertheless, such approaches often rely on user-defined parameter choices and typically yield a single module partition, which may limit their ability to model the multiscale transcriptomic complexity of ASD [20, 21].

To overcome these limitations, Minimum Span Clustering Network (MSCN) was developed as an unsupervised framework for hierarchical gene co-expression analysis without requiring pre-specified thresholds or scale parameters. Rather than optimizing a single network partition, MSCN recursively captures cross-level nearest-neighbor relationships and explicit parent-child module structure [22], thereby tracing transcriptomic signals from fine-grained gene sets to broader biological programs. Unlike lmQCM [23], which allows overlapping co-expression modules, MSCN yields a mutually exclusive, collectively exhaustive (MECE) hierarchy that clarifies module relationships across resolutions. MEGENA likewise provides multiscale network structure [14], but MSCN provides an explicit parent-child module hierarchy that can be followed across levels for downstream interpretation.

Long noncoding RNAs (lncRNAs) are increasingly implicated in ASD and neurodevelopment [24–26]. Autism-specific studies have reported aberrant lncRNA expression in postmortem ASD brain tissue and natural antisense transcripts at ASD-associated loci, including differential expression of *SYNGAP1-AS* [27–29]. Mechanistic studies show that lncRNAs such as *TUNA* and *lnc-NR2F1* regulate neural lineage commitment and neuronal maturation [30, 31]. Integrative analyses of multiple postmortem ASD datasets have also implicated dysregulated lncRNA modules, including *MIR600HG*-linked synaptic ceRNA axes [32], supporting the inclusion of lncRNA dysregulation in ASD transcriptomic network models. Thus, mapping lncRNAs to MSCN-derived mRNA modules offers a practical strategy to link noncoding RNAs to disease-associated modules, especially in the absence of matched lncRNA data across cohorts. By integrating these mRNA modules with lncRNA-to-module mapping and downstream statistics, MSCN facilitates a more comprehensive exploration of ASD-associated transcriptional networks across multiple biological scales from cell-type-specific alterations to broader pathway-level disruptions. Such modular biomarkers and mediation axes could provide a foundation for future diagnostic stratification and targeted therapeutic hypotheses in ASD.

Here, we applied MSCN to two independent postmortem ASD cohorts from cortical regions implicated in social cognition and language processing [27, 33]. We aimed to (1) characterize multilevel co-expression modules spanning from low-level, highly specific patterns to high-level, broadly defined regulatory pathways, (2) explore candidate lncRNA-associated coding-noncoding relationships within ASD-associated mRNA modules, and (3) compare network-level convergences and divergences across datasets. MSCN identified ASD-associated modules enriched for neuronal development, synaptic signaling, and immune processes, thereby supporting recurrent transcriptomic themes previously reported in ASD while extending them within an explicit multilevel architecture. In addition, MSCN prioritized putative transcription factor (TF)-lncRNA-mRNA mediation axes through lncRNA mapping and mediation analysis. These findings support MSCN as a scalable framework for identifying multilevel mRNA co-expression modules and generating testable hypotheses about lncRNA-associated regulatory relationships in ASD and suggest how diverse genetic perturbations may converge within integrated molecular networks to contribute to clinical heterogeneity.

## Materials and methods

### Data collection and covariate assessment

We analyzed two independent cohorts of postmortem brain RNA-seq data. Cohort 1 comprises 167 rRNA-depleted paired-end samples (47 ASD, 44 controls) from the frontal (BA9) and temporal cortex (BA22/41/42), sourced from Parikshak et al. [27]. Following prior evidence that integrating cortical regions enhances detection of shared ASD signatures [27, 34, 35], we merged these areas (85 ASD, 82 controls). Associated metadata included brain region, diagnosis, age, sex, RNA integrity number (RIN), postmortem interval (PMI), batch, and brain bank (see Supplementary Table S1a). Cohort 2 includes 63 prefrontal cortex samples (25 ASD, 38 controls) from Liu et al. [33], with metadata such as diagnosis, age, sex, RIN, tissue source, and sequencing lane (Supplementary Table S1b). To minimize confounding, we assessed major covariates in both cohorts [27, 36]. Pairwise correlations (one-hot encoding) were used to identify interrelated variables, which were then incorporated as fixed effects in a linear mixed-effects (LME) model [27], with individual identity as a random effect. This approach adjusted gene expression values for both technical and biological variability unrelated to ASD [27, 36], providing a robust basis for unsupervised clustering and downstream analyses.

### Module detection using Minimum Span Clustering Network (MSCN)

**Fig. 1:**
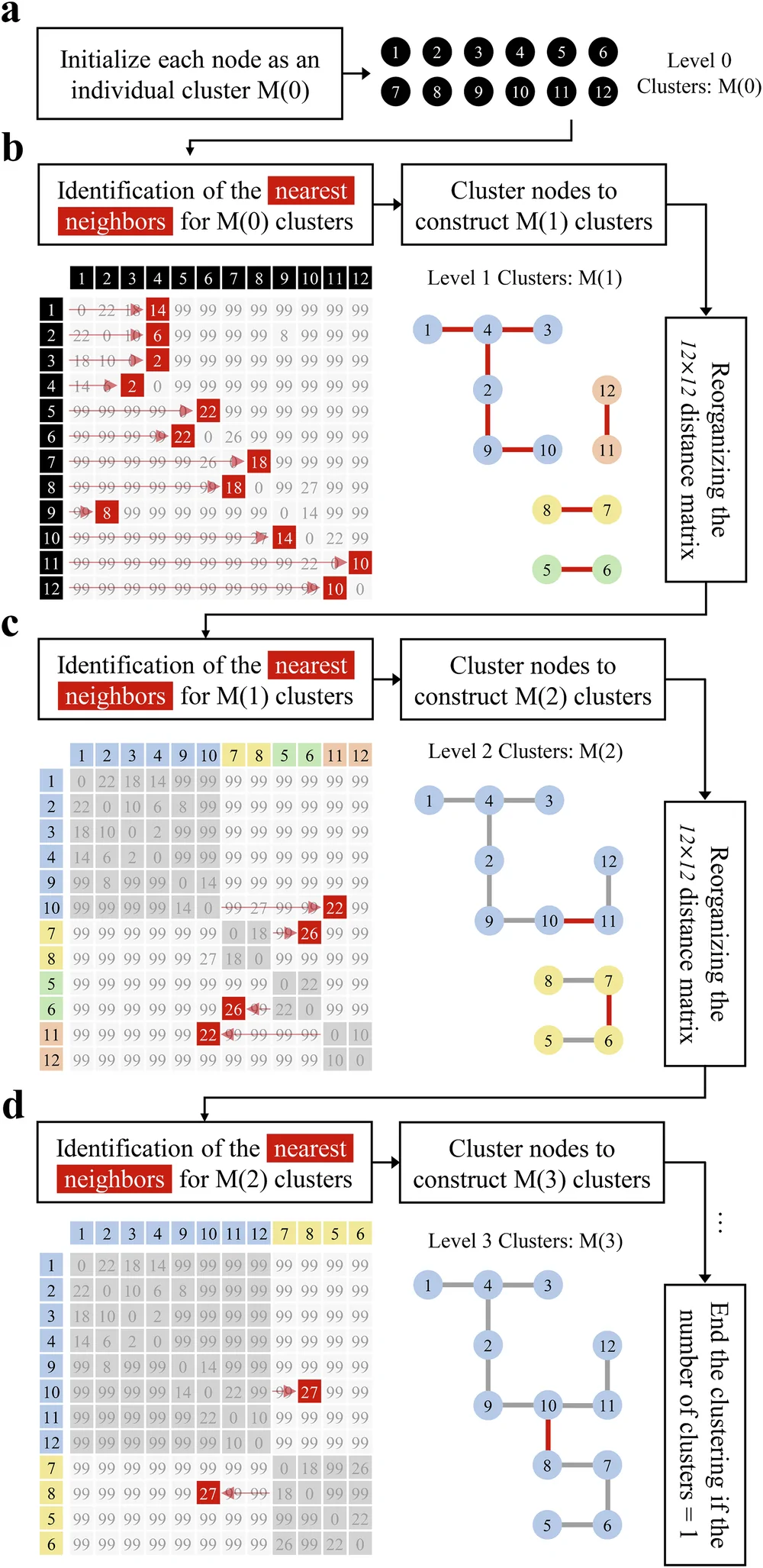
Overview of the MSCN algorithm for multilevel gene co-expression module detection. **a** The MSCN algorithm initializes each gene as an individual node, forming the Level 0 clusters M(0). **b-d** At each iteration, the algorithm identifies the nearest neighbor of each cluster based on the minimum pairwise distance in the symmetric *N* × *N* distance matrix. Clusters connected by nearest-neighbor edges are merged to construct the next-level modules, resulting in a hierarchical module structure: M(1), M(2), …, M(*k*). After each merge, the distance matrix is reorganized according to the current cluster order. **b** At Level 1, clusters are formed by merging the closest individual nodes. **c** At Level 2, previously formed clusters are further merged based on nearest-neighbor relationships. **d** The process continues recursively until the number of clusters reaches 1, indicating that no further merging is possible. Panels **b-d** show a schematic example. For clarity, off-diagonal entries are shown as scaled distances, 100 × *d*_*ij*_, with *d*_*ij*_ = 1 − |*r*_*ij*_|; e.g., 99 denotes 0.99. These values are illustrative and used only to demonstrate nearest-neighbor merging.

We applied the MSCN method, based on minimum span clustering (MSC) [37], to identify co-expression modules. Pairwise Pearson correlations were calculated, and inter-gene distances were defined as *d*_*ij*_ = 1 − |*r*_*ij*_|, where *r*_*ij*_ is the Pearson correlation coefficient between genes *i* and *j*. Smaller off-diagonal distances indicate stronger absolute co-expression. Diagonal entries were set to 0 and excluded from nearest-neighbor searches. Each gene was first considered as an individual module, defined as M(0). Modules at different levels were denoted as M(*i*), where *i* indicates the clustering level. Within each level, individual modules were further numbered sequentially. Thus, a specific first-level module such as the 317th module would be referred to as M(1)_317. This standardized notation provides clear hierarchical and numerical distinctions. The flowchart (Supplementary Fig. S1) and illustrative example (Fig. 1) of MSCN are described below:

Step 1. Initialization: treat each node as an individual cluster (i.e., *N* genes → *N* initial clusters) (Fig. 1a).

Step 2. Clustering: For each cluster, identify and flag the edge(s) (including all edges if they share the same smallest distance) with the minimum distance for merging (Fig. 1b).

Step 3. Reorganization: Merge clusters sharing the shortest edges; update the distance matrix and increase the clustering level (Fig. 1c and d).

Step 4. Multi-Level Clustering: Repeat Steps 2–3 until only one cluster remains, generating a hierarchical modular structure (Fig. 1d).

This iterative clustering generates a multilevel structure of co-expression modules. Only intermediate modules are retained for downstream analysis; the final global cluster is excluded as it lacks internal structure (Supplementary Fig. S2). In this implementation, MSCN has no WGCNA-like grey or unassigned module [20, 38], and requires no soft-thresholding power, pre-specified module number, cut height, or stochastic initialization. Each input mRNA is assigned to one and only one module at each hierarchical level. Modules with fewer than five genes were retained but excluded from downstream eigengene-based association and enrichment analyses because of limited interpretability and unstable eigengene estimation.

### Module-trait associations and functional enrichment

We summarized each MSCN module by its eigengene (first principal component) and computed Pearson correlations with ASD status and covariates in both cohorts [20, 27]. Only modules containing ≥5 genes were analyzed. Multiple comparisons were adjusted by the Benjamini-Hochberg method (FDR < 0.05) [39]. Modules significantly associated with ASD but not with any covariate (*P* > 0.05, one-hot encoding) were classified as differentially expressed modules (DE-modules). Functional enrichment of DE-modules was performed using clusterProfiler (v4.12.6) [40] and org.Hs.eg.db (v3.19.1) [41], focusing on Gene Ontology (GO) Biological Process terms and Kyoto Encyclopedia of Genes and Genomes (KEGG) pathways.

### Prediction of lncRNA-mRNA associations

We included 1770 lncRNAs (lincRNAs, processed transcripts, antisense RNAs) from Cohort 1 [27], with covariate adjustment as above. Because lncRNAs were quantified only in Cohort 1, they were excluded from initial module construction. Instead, Cohort 1 lncRNAs were mapped to MSCN-derived Level 1 mRNA modules based on nearest-neighbor expression correlations without re-clustering. Multiple memberships and unassigned lncRNAs were not modeled in the current implementation. This mapping was used to annotate cohort-comparable mRNA modules and to define candidate lncRNAs for downstream mediation analysis.

### Identification of upstream regulators and mediation analysis in ASD-related modules

Upstream regulators of ASD-related modules were identified using Enrichr (v3.4) with the “TRANSFAC_and_JASPAR_PWMs” database [42]; only TFs present within the module (adjusted *P* < 0.05) were considered for further analysis. Mediation analysis (mediation package v4.5) [43] was applied to assess whether lncRNAs mediated the effects of these TFs on target mRNA expression. An lncRNA was defined as a significant mediator if: (i) the average direct effect (ADE) *P* ≥ 0.05, (ii) both average causal mediation effect (ACME) and total effect *P* < 0.05, and (iii) the directions of ACME and total effect were consistent.

## Results

### Multilevel gene co-expression modules revealed by MSCN

**Fig. 2:**
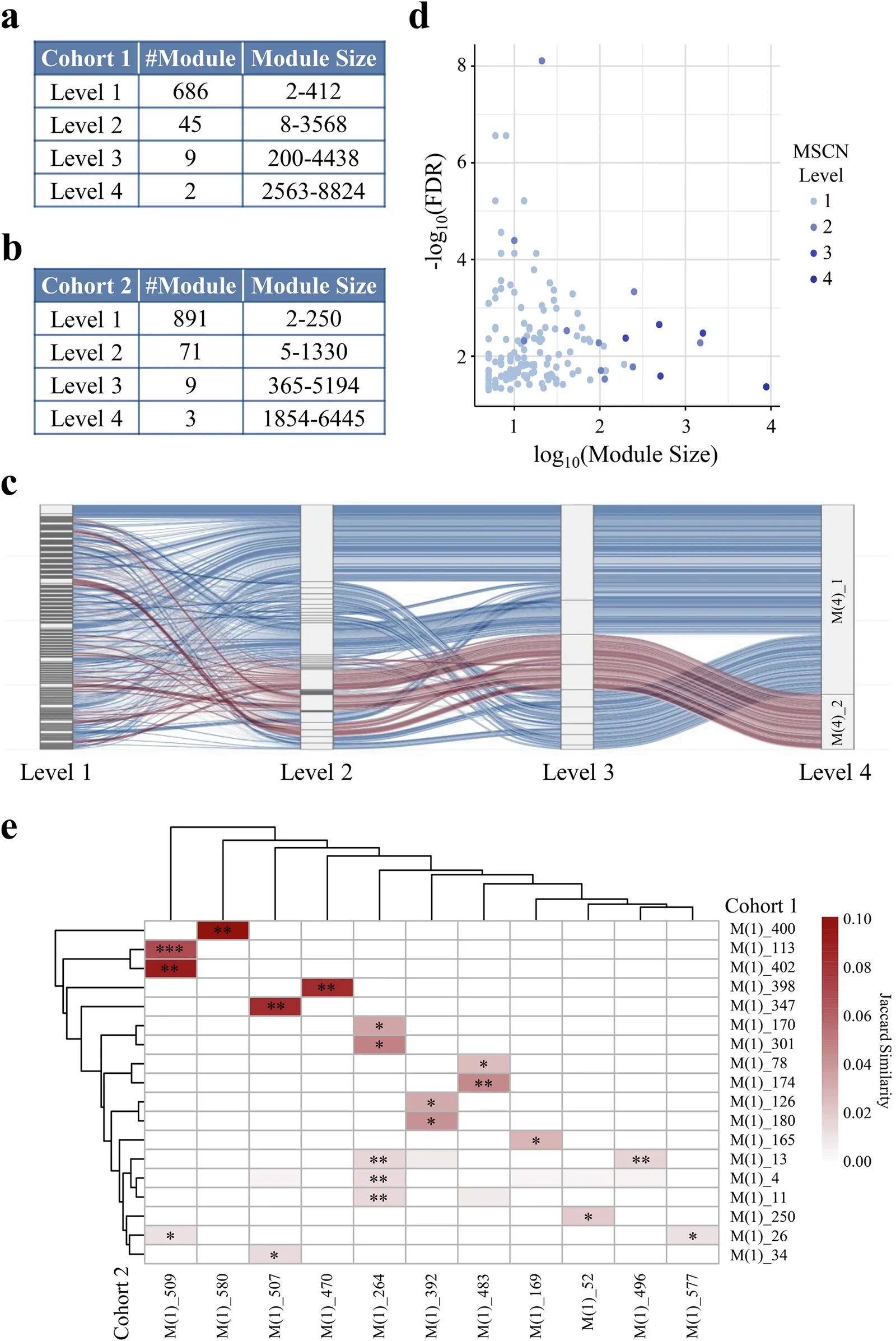
Hierarchical co-expression modules identified by MSCN in two ASD brain transcriptome cohorts. **a,** **b** Summary of MSCN module decomposition across four hierarchical levels in Cohort 1 **a** and Cohort 2 **b**. The number of modules (#Module) decreases with increasing levels, reflecting the progressive merging of co-expression clusters. Correspondingly, module sizes expand, capturing broader transcriptional structures at higher levels. **c** Alluvial plot representing MSCN module merging across Levels 1 to 4 in Cohort 1. **d** Relationship between module size and ASD-association significance among DE modules in Cohort 1. Each point represents a module; color indicates MSCN level (Levels 1 to 4, light to dark blue). **e** Cross-cohort reproducibility of ASD-enriched modules. Heatmap shows the Jaccard similarity of DE module compositions between Cohort 1 and Cohort 2 at MSCN Level 1. Only modules that could be matched to at least one module in the other cohort (i.e., with non-zero Jaccard similarity) were retained for visualization. Significant overlaps are marked by asterisks, denoting hypergeometric *P* values: *P < 0.05, **P < 0.01, ***P < 0.001.

To dissect ASD transcriptomic complexity, we applied the MSCN algorithm to identify gene co-expression modules across multiple hierarchical levels (see Methods, Fig. 1, and Supplementary Fig. S1). We first examined potential technical and biological confounders in both cohorts to ensure that the resulting gene modules reflect ASD-associated transcriptional patterns rather than batch effects or sample heterogeneity. For Cohort 1, covariates included brain region, diagnosis, age, sex, RIN, PMI, sequencing batch, and brain bank. For Cohort 2, we considered diagnosis, age, sex, RIN, tissue source, and sequencing lane. Pairwise correlation analyses using one-hot encoding revealed substantial covariate interdependencies (Supplementary Fig. S3a and b). We therefore applied a linear mixed-effects (LME) model to regress out variation unrelated to ASD diagnosis, using individual sample identity as a random effect and all other variables as fixed effects. This expression-adjustment step enhanced the signal-to-noise ratio in downstream analyses and ensured that the clustering results reflected disease-relevant molecular architecture. We then applied MSCN to the adjusted expression data from 11,387 genes shared between cohorts. MSCN recursively merged modules based on shortest inter-gene distances, resulting in a four-level modular hierarchy of M(1) through M(4) for both cohorts. In Cohort 1, the method identified 686 fine-grained modules at M(1), which merged into 45 broader modules at M(2), nine modules at M(3), and two highest-level clusters at M(4) (Fig. 2a). Module sizes ranged from 2 to 8824 genes, revealing structured transcriptional patterns at multiple scales (Supplementary Table S2a). Cohort 2 exhibited a similar multilevel structure, with 891 modules at M(1), 71 at M(2), nine at M(3), and three at M(4) (Fig. 2b and Supplementary Table S2b). This consistency across datasets demonstrates the reproducibility of MSCN and supports its utility in capturing layered gene expression relationships in ASD. To further illustrate the hierarchical structure identified by MSCN, we plotted the parent-child relationships among Cohort 1 modules across Levels 1–4 (Fig. 2c). This flow diagram shows how many fine-grained Level 1 modules are progressively merged through intermediate Level 2 and Level 3 modules into two broad Level 4 modules, M(4)_1 and M(4)_2, forming a directly traceable hierarchy.

To identify ASD-associated modules, we computed module eigengenes (MEs) and tested their correlation with ASD diagnosis using Pearson’s r and Benjamini-Hochberg (BH) correction. In Cohort 1, we detected 135, 10, 4, and 1 differentially expressed (DE) modules at M(1), M(2), M(3), and M(4), respectively (FDR < 0.05) (Fig. 2d and Supplementary Table S3a). Although module size increased at higher MSCN levels due to hierarchical merging, ASD association significance did not show a simple monotonic relationship with module size. Significant modules were observed across multiple scales, and the strongest association was observed for the intermediate Level 2 module M(2)_34, whereas larger higher-level modules showed more moderate significance. In Cohort 2, stronger correlations between diagnosis and covariates such as RIN and tissue source likely obscured ASD-related signals (Supplementary Fig. S3b). Therefore, to avoid overcorrection [20, 44], we identified nominal ASD-associated modules based on unadjusted *P* values, resulting in 17 modules at M(1) and one module at M(2) (Supplementary Table S3b and Fig. S4). Importantly, these modules showed no significant associations with confounders (all uncorrected *P* values > 0.05), suggesting that their expression patterns were related to ASD rather than technical or biological artifacts (Supplementary Table S3 and Figs. S5 and S6). Cross-cohort comparison of ASD-associated modules revealed complementary patterns across MSCN levels. At Level 1, we observed widespread but distributed overlaps: several modules from Cohort 1 (e.g., M(1)_400, M(1)_113, M(1)_402) significantly intersected with distinct modules from Cohort 2 (Fig. 2e), indicating partial preservation of specific transcriptional architecture. At Level 2, although only a single ASD-associated module was identified in Cohort 2 (M(2)_67), it exhibited significant overlap with three Level 2 modules from Cohort 1 (M(2)_1, M(2)_30, and M(2)_34; hypergeometric *P* values = 0.044, 0.032, and 0.018, respectively). Our findings show that MSCN consistently captures multiscale modular structures across two independent ASD datasets. These cross-cohort overlaps were used as evidence of module preservation rather than as the primary substrate for TF-lncRNA-mRNA mediation analysis, because Level 1 cross-cohort matches were often distributed rather than one-to-one and matched lncRNA profiles were unavailable in Cohort 2.

### Comparison with WGCNA and MEGENA supports the multiscale structure of MSCN

To compare MSCN with established co-expression approaches, we applied WGCNA and MEGENA to the same covariate-adjusted Cohort 1 mRNA expression matrix used for MSCN (Supplementary Methods). WGCNA recovered the broad ASD-associated co-expression signal, with several MSCN DE-modules showing significant overlap with the ASD-significant WGCNA blue module, indicating that WGCNA captures an overarching ASD module but represents multiple MSCN levels within a single flat partition (Supplementary Fig. S7). MEGENA also recovered ASD-associated co-expression structures overlapping MSCN modules (Supplementary Fig. S8a); however, these signals were often represented as multiple smaller MEGENA modules within the same MSCN parent-module context, consistent with a more fragmented representation of broader MSCN modules (Supplementary Fig. S8b). We therefore view MSCN as a complement to WGCNA and MEGENA since it provides a deterministic, directly traceable parent-child hierarchy that facilitates downstream module-level interpretation.

In addition, we performed a method-level comparison among MSCN, WGCNA, and MEGENA using module preservation, enrichment strength, and runtime. Across module levels and size categories, MSCN showed preservation and enrichment-strength distributions comparable to established methods, indicating that the MSCN hierarchy did not sacrifice cross-cohort reproducibility (Supplementary Fig. S9a-e). Runtime benchmarking was performed on the same covariate-adjusted Cohort 1 mRNA expression matrix and measured clustering/module construction only. WGCNA was the fastest implementation in this benchmark, completing module construction in 11 s, whereas the R-based MSCN implementation completed in 95 s and MEGENA required 1790 s (Supplementary Fig. S9f). Thus, MSCN was approximately 18.8-fold faster than MEGENA while providing an explicit, directly traceable multilevel hierarchy (Fig. 2c), although it was slower than the single-partition WGCNA workflow. Together, these comparisons support MSCN as a computationally practical and biologically concordant framework that complements existing co-expression methods by providing deterministic multilevel module structure.

### Functional enrichment of ASD-associated modules

**Fig. 3:**
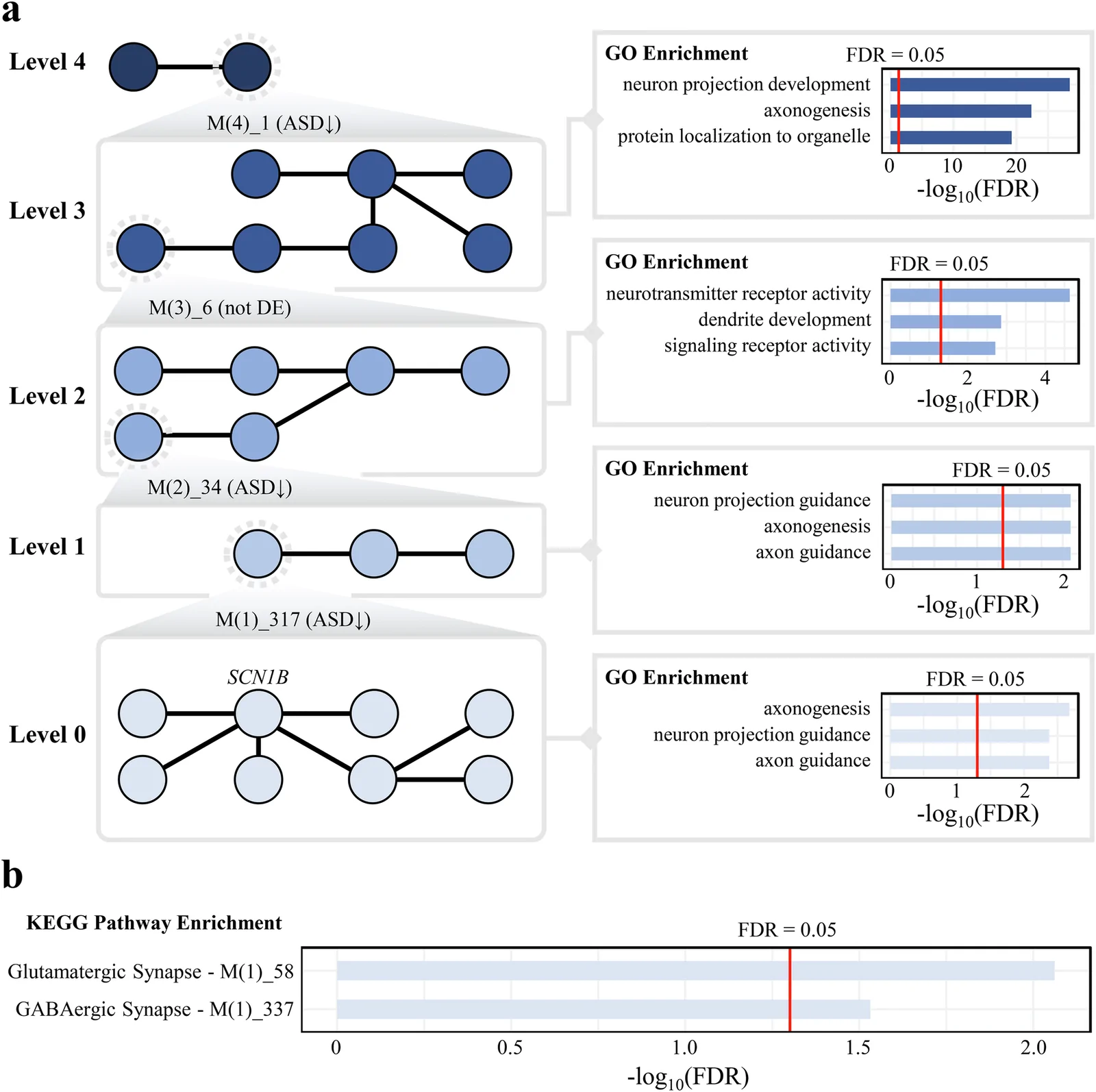
Multilevel functional preservation and convergence of ASD-associated gene modules identified by MSCN. **a** Hierarchical representation of module M(2)_34 and related sub- and super-modules in MSCN. Module identifiers and ASD eigengene directions (ASD↑/↓) are shown beside each module; modules included only for hierarchy illustration are labeled as “not DE.” Gene Ontology enrichment analysis reveals conserved neurodevelopmental functions across levels, including axon guidance, dendrite development, and neurotransmitter signaling. Enrichment significance is indicated by -log_10_(FDR) on the bar plots. **b** Additional DE-modules show significant enrichment for KEGG pathways, including glutamatergic synapse (M(1)_58) and GABAergic synapse (M(1)_337).

Having established that MSCN recovered reproducible and methodologically concordant multilevel modules, we next examined whether ASD-associated modules corresponded to interpretable biological processes. To assess the biological relevance of MSCN-derived co-expression modules, we performed Gene Ontology (GO) and KEGG pathway enrichment analyses across all ASD-associated modules (Supplementary Table S3). As a representative case (Fig. 3 and Supplementary Table S3), we highlight module M(2)_34—the most significantly ASD-associated module in Cohort 1 (FDR = 7.78 × 10^−9^). This module and its higher- and lower-level relatives form a well-aligned neurodevelopmental axis across the MSCN hierarchy. Specifically, its parent module M(3)_6 and grandparent module M(4)_1 are enriched for key neuronal processes including neuron projection development, axonogenesis, neurotransmitter receptor activity, dendrite development, and signaling receptor activity (all FDR < 0.01; Fig. 3a). At the lowest level, M(1)_317—contained within M(2)_34—exhibited a highly functionally consistent enrichment profile (axonogenesis, axon guidance, and neuron projection guidance), supporting cross-level functional consistency (Supplementary Table S3). Within M(1)_317, the hub gene *SCN1B* (five edges) is of particular interest, as it has been linked to seizure disorders such as epilepsy and Dravet syndrome [45, 46], both of which show clinical overlap with ASD. The consistency across levels suggests that MSCN not only captures clustering patterns ranging from specific gene co-expression to broader biological pathways but also aligns these multiscale structures with known ASD-relevant neurodevelopmental processes. As complementary examples, additional Cohort 1 DE modules also revealed ASD-related biological processes (Supplementary Table S3). For instance, while M(3)_1 (descended from M(4)_1) was not directly associated with ASD, it exhibited strong KEGG enrichment in Parkinson’s disease pathways (FDR = 1.4 × 10^−21^), indicating its involvement in broader neuronal functions beyond ASD. Furthermore, the Level 1 modules—M(1)_58 and M(1)_337—were significantly enriched in glutamatergic and GABAergic synapse pathways, respectively (FDR = 0.0087 and 0.029) (Fig. 3b and Supplementary Table S3). Our findings demonstrate that MSCN can resolve hierarchically embedded modules with distinct functions, even when part of the same top-level co-expression structure. These observations underscore the modular complexity underlying ASD and highlight the divergent functional specializations within the same higher-level cluster. Although no modules in Cohort 2 passed the FDR threshold due to confounding influences, several Level 1 modules showed suggestive functional relevance. Notably, M(1)_422 (unadjusted *P* value = 0.02) was enriched for *in utero* embryonic development (FDR = 0.002), while M(1)_580 (unadjusted *P* value = 0.04) mapped to the Adherens junction KEGG pathway (FDR = 0.01) (Supplementary Table S3). These annotations point to potential involvement in early neurodevelopmental or cell adhesion processes, which suggests the need for further investigation in larger datasets. Although these nominal ASD-associated modules did not reach statistical significance for ASD (based on FDR), their functional annotations suggest biological processes that require additional research in the context of neurodevelopmental disorders. Across hierarchical levels and cohorts, our results support the validity of MSCN as a biologically informative framework and underscore the potential of modular co-expression analysis to reveal cryptic structure in complex transcriptomic landscapes.

### Functional associations and candidate TF-lncRNA-mRNA axes in ASD modules

**Fig. 4:**
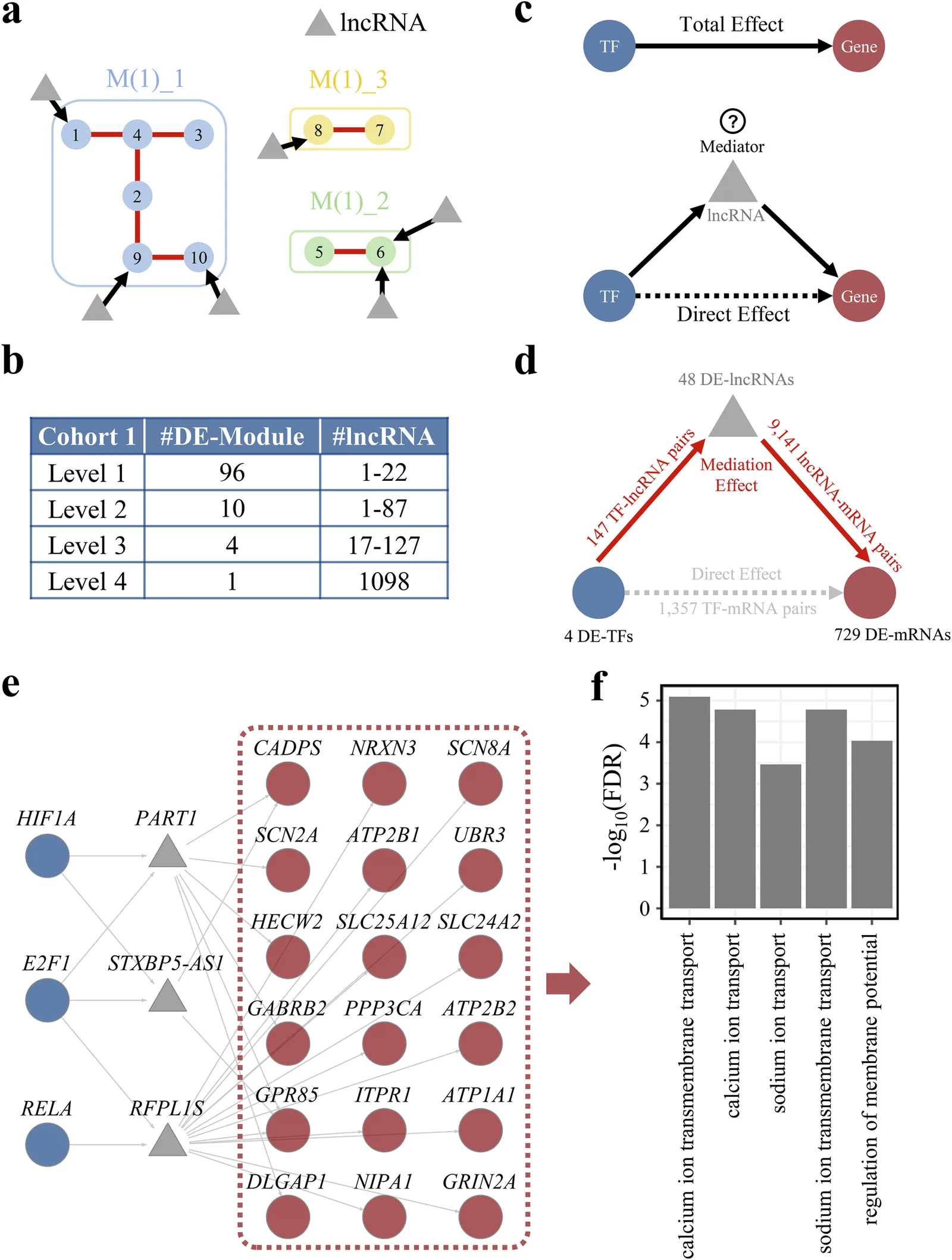
Mapping of lncRNAs and candidate TF-lncRNA-mRNA mediation analysis within MSCN-derived ASD modules. **a** Schematic representation of how lncRNAs were assigned to MSCN Level 1 mRNA modules through nearest-neighbor co-expression relationships. Gray triangles denote lncRNAs, and colored boxes highlight distinct modules. **b** A summary table shows the number of DE-modules at each MSCN level containing at least one mapped lncRNA. **c** Conceptual overview of the mediation analysis framework. The total effect of a transcription factor (TF) on a target mRNA is decomposed into a direct effect and an indirect effect mediated by the lncRNA. **d** Summary statistics for the 11,252 significant TF-lncRNA-mRNA mediation axes. **e** Named lncRNA-SFARI subnetwork constructed from the 789 externally supported axes. **f** Top five GO enrichment terms of 18 SFARI target genes in panel **e**.

To explore lncRNAs associated with ASD-related mRNA modules, we mapped lncRNAs to MSCN-derived mRNA modules in Cohort 1. Rather than clustering lncRNAs separately, we assigned them to their nearest-neighbor mRNA modules based on correlation proximity in the lowest-level network (Fig. 4a). Across all hierarchical levels, we identified ASD-associated modules with at least one mapped lncRNA at each level: 96 at M(1), 10 at M(2), 4 at M(3), and 1 at M(4) (Fig. 4b; Supplementary Table S4). Among these, module M(1)_56 emerged as particularly notable since 22 lncRNAs were mapped to this module, which was enriched for glycerophospholipid metabolism (FDR = 0.0096), aligning with prior biochemical findings in autism, wherein altered levels of phosphatidylethanolamine and phosphatidylserine were reported in patient erythrocyte membranes and plasma, suggesting a potential metabolic signature linked to ASD pathophysiology [47]. These findings suggest that mapped lncRNAs are co-expressed with specific functional mRNA modules and may represent candidates for downstream regulatory hypothesis testing. To further explore the potential regulatory architecture of ASD transcriptomes, we extended our network analysis to infer upstream TFs and potential mediation roles of lncRNAs. We first used the Enrichr platform and the “TRANSFAC_and_JASPAR_PWMs” database to identify TFs enriched among genes in ASD-related modules (Supplementary Table S5a). We identified 11 ASD-associated M(1) modules, 4 M(2), 3 M(3), and 1 M(4) modules with at least one enriched TF. To ensure biological relevance in the current clustering results, we limited the analysis to TFs present in the modules. Notably, TFs were detected in M(2)_2, M(3)_2, and M(4)_1 modules, with 5, 6, and 15 TFs, respectively. As shown in the cross-method comparison above, the three modules were also concordant with the WGCNA modules most significantly associated with ASD at each MSCN level (Supplementary Fig. S7), supporting their use for downstream analysis. We therefore focused on these high-level ASD-associated modules to identify candidate upstream TFs and TF-lncRNA-mRNA mediation axes.

To assess whether lncRNAs statistically mediate TF-mRNA expression associations, we applied a statistical mediation analysis framework. We asked whether lncRNAs act as mediators between TFs and their candidate target mRNAs (Fig. 4c). Within M(4)_1, we focused on DE-TFs, DE-lncRNAs, and DE-mRNAs (differential gene-expression statistics for ASD were obtained from Parikshak et al. [27]), resulting in 4 TFs, 51 lncRNAs, and 822 mRNAs, which formed 167,688 candidate TF-lncRNA-mRNA axes. Mediation analysis revealed that 11,252 (6.7%) of these axes passed the significance threshold (*P*_ACME_ < 0.05, *P*_Total_ < 0.05, *P*_ADE_ ≥ 0.05, and the directions of ACME and total effect estimates were consistent; Supplementary Table S5b). This statistical pattern is consistent with a candidate mediation model in which lncRNA expression accounts for part of the TF-mRNA association. Among the significant mediation axes, the unique nodes comprised 4 DE-TFs, 48 DE-lncRNAs, and 729 DE-mRNAs, corresponding to 147 TF-lncRNA pairs, 9141 lncRNA-mRNA pairs, and 1357 TF-mRNA pairs (Fig. 4d). SFARI annotation of the 11,252 mediation axes identified 644 TF-lncRNA-mRNA axes involving 46 SFARI genes with scores of 1 or 2, including four syndromic genes. These axes involved 12 named lncRNAs as candidate mediators, offering a focused yet expansive resource for future biomarker development and mechanistic exploration in ASD (Supplementary Table S5b). To contextualize these candidate mediators, we further curated all 12 named lncRNAs in Supplementary Table S5c, summarizing their evidence and reported relevance to brain, ASD, and/or neuropsychiatric biology.

To evaluate external support for the 11,252 mediation axes, we tested the axes that were measurable in external ASD-related transcriptomic resources [48] (Supplementary Methods). Among these axes, 10,459 were externally testable, and 789 satisfied the same mediation criteria with concordant effect directions (Supplementary Table S5b). To explore the functional implications of the externally supported lncRNA layer, we further filtered the 789 externally supported axes to retain axes involving three named lncRNAs and 18 SFARI target genes (Fig. 4e). The 18 SFARI target genes retained in this subnetwork were enriched for calcium ion transmembrane transport, calcium ion transport, sodium ion transport, sodium ion transmembrane transport, and regulation of membrane potential (Fig. 4f). These enrichments suggest that the externally supported lncRNA-associated axes may converge on neuronal excitability and ion-transport processes relevant to ASD. Within this externally supported SFARI-related subnetwork, we highlighted three axes to illustrate the biological relevance of the lncRNA layer: *HIF1A-STXBP5-AS1-CADPS*, *E2F1-PART1-SCN2A*, and *RELA-RFPL1S-GRIN2A* (Fig. 4e). The first representative axis, *HIF1A-STXBP5-AS1-CADPS*, suggests a potential lncRNA-mediated mechanism linking transcriptional regulation to neurodevelopmental processes. *HIF1A*, a key TF responsive to hypoxia and inflammation, was significantly activated in the frontal cortex of ASD model mice, particularly in microglia, suggesting a potential role in the neuroimmune dysregulation underlying ASD, as supported by its differential expression in ASD compared to controls (log₂ fold change = 0.247; FDR = 0.048; Supplementary Table S5b) [49]. *STXBP5-AS1* is a long non-coding RNA associated with Attention Deficit Hyperactivity Disorder (ADHD) symptom severity in both adults and children, suggesting that *STXBP5-AS1* may be relevant to synaptic function and ASD (log₂ fold change = −0.457; FDR = 0.012; Supplementary Table S5b) [50]. *CADPS*, a gene involved in calcium-dependent vesicular exocytosis and synaptic plasticity, may be relevant to ASD due to its critical role in neuronal signaling (log₂ fold change = −0.303; FDR = 0.033; Supplementary Table S5b) [51]; notably, it is also listed in the SFARI Gene database [1, 52]. Mediation analysis revealed that *STXBP5-AS1* significantly mediated the effect of *HIF1A* on *CADPS* (*P*_ACME_ < 0.001; *P*_ADE_ = 0.116; 55.4% mediated), indicating a candidate lncRNA-associated mediation axis (Supplementary Table S5b).

We further highlighted two additional representative mediation axes, *E2F1-PART1-SCN2A* and *RELA-RFPL1S-GRIN2A*. We first focused on the *E2F1-PART1-SCN2A* axis, in which *PART1* was selected because it has been implicated in neurological contexts, including Parkinson’s disease-related lncRNA/ceRNA analyses and MPP + -induced neuronal injury in SH-SY5Y cells [53, 54]. Within this axis, *E2F1* is implicated in neuronal apoptosis and age-dependent synaptic/behavioral phenotypes [55, 56], whereas *SCN2A* encodes the NaV1.2 sodium channel and is a high-confidence ASD/neurodevelopmental risk gene [57, 58]. We then turned to the *RELA-RFPL1S-GRIN2A* axis as a second example. *RFPL1S* shows strong brain-enriched expression, including in the amygdala and prefrontal cortex [59], and has been reported as a hub lncRNA in a neuron-related amygdala co-expression module in schizophrenia, with associated pathways including axon, synapse, and dendrite [60]. Within this axis, RELA/NF-κB signaling is relevant to neuronal excitatory/inhibitory regulation, synaptic plasticity, and ASD-related neuroinflammation [61–63], whereas *GRIN2A* encodes an NMDA receptor subunit associated with epilepsy, speech/language impairment, neurodevelopmental and neuropsychiatric phenotypes [64]. Importantly, external transcriptomic data further supported the mediation relationships underlying all three representative axes (Supplementary Table S5b). These findings support potential coding-noncoding relationships in ASD, whereby lncRNAs may mediate TF-associated expression relationships linked to synaptic or metabolic genes. This multi-layered network highlights the complex interplay between coding and noncoding transcripts in ASD and offers a conceptual framework for future experimental validation.

## Discussion

This study presents MSCN, an unsupervised hierarchical clustering method that provides a deterministic, directly traceable multilevel module hierarchy. Unlike standard WGCNA workflows, MSCN does not require selection of a soft-thresholding power or a tree-cutting threshold during module construction. Because lncRNA data were unavailable in one cohort, we constructed the primary MSCN hierarchy using mRNAs and then mapped Cohort 1 lncRNAs onto the mRNA modules; this design can be extended to other RNA types (e.g., circRNAs, microRNAs) [34, 36, 65]. We identified ASD-associated modules enriched for neurodevelopmental, synaptic, and immune pathways [9], as well as candidate lncRNA-associated mediation axes, expanding current models of ASD pathogenesis. Mediation analysis revealed that ~6.7% of candidate TF-lncRNA-mRNA axes satisfied the mediation criteria, suggesting potential functional roles for noncoding RNAs in ASD (Fig. 4 and Supplementary Table S5). Integration with SFARI gene annotations highlighted 644 TF-lncRNA-mRNA axes involving 46 high-confidence ASD risk genes and 12 named lncRNA mediators, providing convergent evidence of their potential roles. Notably, we identified several axes, including *HIF1A-STXBP5-AS1-CADPS*, *E2F1-PART1-SCN2A*, and *RELA-RFPL1S-GRIN2A*, with mediation proportions exceeding 50%. These results nominate the corresponding lncRNAs as statistically supported candidate mediators of ASD-related gene expression patterns and generate testable hypotheses for future validation. Because these analyses are based on observational bulk transcriptomic data, they cannot distinguish direct molecular regulation from indirect effects, reverse causality, pleiotropy, or cell-type composition differences. Functional perturbation experiments will be required to establish causal regulation.

The hierarchical MSCN framework revealed functional modules aligned with established ASD mechanisms. At the highest level (M(4)), modules were strongly enriched for neuronal projection development (FDR = 3.47 × 10^−29^), consistent with reports of altered dendritic spine density in ASD (Fig. 3a and Supplementary Table S3) [66]. Lower levels identified modules involved in oligodendrocyte differentiation, synapse maturation, axonogenesis, and immune pathways, reflecting the heterogeneity of ASD pathology [67]. The proportion of ASD-associated modules showing significant SFARI enrichment increased from 17.8% at lower levels to 100% at the highest level (hypergeometric test *P* < 0.05), demonstrating that broader, higher-order modules capture key ASD genes and may serve as candidate biomarkers. These results support the biological relevance of MSCN’s multiscale clustering and illustrate how broad functional modules subdivide into disease-relevant pathways. This modular organization not only clarifies ASD transcriptomic architecture but also provides gene sets that could support future biomarker development in accessible tissues such as blood or cerebrospinal fluid to improve the precision of diagnostics and therapeutic monitoring.

To clarify the data conditions under which MSCN performs most robustly, we performed sensitivity analyses using Cohort 1 (Supplementary Methods). ASD-associated modules remained detectable after FDR correction in repeated down-sampling analyses, even when the sample size was reduced to 60 (Supplementary Fig. S10a). Additional analyses of covariate imbalance showed a clear reduction in the number of FDR-significant modules as brain-region imbalance increased, whereas sequencing-batch imbalance produced condition-dependent and non-monotonic changes (Supplementary Fig. S10b). These findings suggest that sample size alone does not fully account for the lack of FDR-significant modules in Cohort 2. Rather, FDR-controlled module-trait inference is most sensitive to the combined effects of reduced effective sample size, diagnosis-covariate collinearity, and tissue/source heterogeneity. Thus, MSCN can be used to recover multilevel co-expression architecture in smaller datasets, but confirmatory phenotype-associated module detection is most reliable when samples are sufficiently balanced across major technical and biological covariates and when tissue/cell-type heterogeneity is limited or appropriately adjusted.

### Methodological strengths and comparisons

MSCN complements existing clustering methods by reducing parameter selection during module construction and preserving traceable hierarchical gene relationships, thereby reducing the multiple testing burden relative to gene-wise association studies [20]. MSCN builds on the MSC framework, which has shown robust clustering performance in prior biological similarity-network applications [68, 69], while maintaining computational scalability (e.g., 20,000 genes in ~450 s; Supplementary Fig. S11) and providing a deterministic multilevel structure for gene co-expression analysis. Its theoretical basis, converging to a minimum spanning tree when gene distances are unique, ensures interpretability and algorithmic integrity [70, 71]. Additionally, MSCN allows flexible integration with other approaches and supports correlation thresholding to minimize spurious associations. Empirically, an absolute correlation threshold of approximately 0.6 separated stronger intra-module edges from weaker inter-cluster edges in these datasets (Supplementary Fig. S12) and may serve as a heuristic for prioritizing higher-confidence edges for downstream analyses (for example, the number of lncRNAs in M(4)_1 decreases from 1098 to 660), where biologically meaningful edges are expected to display stronger co-expression. In the absence of gold-standard benchmarks, we validated MSCN-derived modules using a multilayered strategy. First, we observed reproducible hierarchical structures and overlapping ASD-associated modules across two independent cohorts (Fig. 2; Supplementary Fig. S4; Supplementary Table S3). Second, functional enrichment analyses consistently identified neurodevelopmental, synaptic, and immune pathways, while module eigengenes correlated with diagnosis independent of confounders, confirming specificity to ASD (Fig. 3; Supplementary Table S3; Supplementary Figs. S5 and S6). SFARI gene enrichment increased with module hierarchy, indicating that broader modules capture key ASD risk signatures (Supplementary Table S3). Additionally, network topology analyses revealed a correlation threshold (|*r*| = 0.6) that distinguished stronger co-expression edges from weaker inter-cluster edges (Supplementary Fig. S12). Finally, to assess whether MSCN can recover known module structure, we generated simulated expression matrices with ground-truth module labels using WGCNA’s built-in simulation utility (Supplementary Methods). Across 50 independent replicates in this WGCNA-generated flat-module setting, MSCN and WGCNA showed comparable ARI values, while MSCN achieved significantly higher NMI and a numerically higher median ARI (Supplementary Fig. S13). These results suggest that MSCN preserves exact module recovery comparable to WGCNA while better retaining overall clustering information in a WGCNA-aligned flat-module scenario. We therefore interpret this analysis as a conservative benchmark indicating that MSCN is positioned as a complementary multilevel framework rather than a universal replacement for WGCNA. Its key advantage lies not in universal superiority, but in the deterministic construction of a directly traceable parent-child module hierarchy (Fig. 2c). Together, these convergent lines of evidence establish the reliability and biological relevance of the MSCN framework.

### Limitations and considerations

While MSCN offers key methodological advances, several limitations remain. First, gene-level expression from heterogeneous tissues may obscure cell-type- or isoform-specific co-expression, and cohort-specific confounders may reduce the power to detect ASD modules under stringent correction (Supplementary Figs. S3 and S6). Replication in larger, homogeneous datasets will be necessary to confirm disease relevance. Second, similar to standard non-grey WGCNA modules, module membership in MSCN is non-overlapping at each level. Although its hierarchical structure captures nested organization across scales, it does not explicitly model genes, TFs, or lncRNAs participating in multiple overlapping functional programs [72]. Consequently, pleiotropic or boundary genes may be represented only by their dominant co-expression context at a given level, potentially understating overlap among ASD-related neuronal, developmental, and immune modules. The same consideration applies to TF-lncRNA-mRNA mediation axes, which should be interpreted as module-anchored, dominant co-expression relationships rather than exhaustive representations of all cross-module regulatory roles. Future extensions could incorporate soft or overlapping memberships or compare MSCN with overlapping community-detection methods [72]. Third, causal inference from observational data is fundamentally limited: mediation analysis cannot distinguish true causal mediation from pleiotropy or reverse causation, as Markov equivalence precludes definitive directionality without temporal or perturbational data (e.g., knockdown or overexpression) [73]. We underscore that any inferred directions should be interpreted with caution and ideally supported by further evidence before drawing definitive causal conclusions. Notably, our approach still reduces over 90% of candidate pairs, thereby significantly reducing the burden of subsequent analyses. Fourth, lncRNAs were mapped to MSCN-derived mRNA modules rather than included in the initial clustering because matched lncRNA quantification was available only in Cohort 1, whereas the primary mRNA hierarchy was built from genes shared across both cohorts. This preserved cross-cohort comparability and provided a practical framework for downstream mediation analysis. However, we did not directly benchmark this approach against joint mRNA-lncRNA clustering. Thus, our results should not be taken as evidence that post hoc lncRNA mapping better captures regulatory relationships than joint clustering. Future directions include integrating MSCN with graph-based machine learning, functional validation (e.g., CRISPR-based gene editing or antisense oligonucleotide interventions), and clinical expansion to multi-omics and larger cohorts to enable precision psychiatry.

## Conclusion

This study presents MSCN as an unsupervised framework for mapping multilevel mRNA co-expression architecture in ASD. MSCN reveals hierarchical co-expression patterns that reflect distinct biological programs, including neurodevelopmental, synaptic, and immune processes. By mapping lncRNAs to MSCN-derived mRNA modules in Cohort 1, we identified candidate lncRNA-associated TF-mRNA mediation axes, offering network-level hypotheses of transcriptional regulation in ASD for future validation. These findings deepen our systems-level understanding of ASD pathophysiology and highlight modular biomarkers with potential diagnostic or therapeutic relevance. As a scalable and generalizable framework, MSCN can be extended to other neuropsychiatric disorders and omics layers, providing a flexible tool for dissecting transcriptional regulation in complex disease contexts.

## Supplementary information

Supplementary Information

Table S1

Table S2

Table S3

Table S4

Table S5

## Data Availability

The original input datasets used in this study are publicly available. Cohort 1 was obtained from Synapse (syn4587609), and Cohort 2 was obtained from GEO (GSE51264 and GSE59288) and the associated published supporting data. The processed data generated in this study and required to reproduce the reported results, including covariate-adjusted expression matrices, module assignments, edge lists, module-level statistics, enrichment results, lncRNA-module mappings, and TF-lncRNA-mRNA mediation outputs, are fully provided in the main text or supplementary materials.

## Code availability

The source code for MSCN is publicly available on GitHub (https://github.com/telunmai/MSCN).
