# Supplementary material for: Mapping lncRNAs onto multilevel mRNA co‑expression modules in autism
Source: Transl Psychiatry. 2026 Jul 13;16:547. doi: 10.1038/s41398-026-04275-0 (PMC13652821; doi:10.1038/s41398-026-04275-0)
Supplement: Supplementary file 1 [file 41398_2026_4275_MOESM1_ESM.pdf]

SUPPORTING INFORMATION

Mapping lncRNAs onto multilevel mRNA co-expression modules in autism

Chen-Ling Lee[1], Geng-Ming Hu[2], Yi-Pei Li[1], and Te-Lun Mai[1,3*]

[1]Department of Life Science, National Taiwan University, Taipei, Taiwan
[2]Department of Physics, National Taiwan Normal University, Taipei, Taiwan
[3]Center for Computational and Systems Biology, National Taiwan University, Taipei, Taiwan
*Correspondence and requests for materials should be addressed to T.-L.M. (tlmai@ntu.edu.tw)

Supporting Information Overview
This supplementary file provides additional materials supporting the results presented in the main manuscript. It includes:

[1] Supplementary Methods (Pages S2-S3)
This section describes additional analytical procedures used for cross-method comparison, robustness assessment, and external support analyses. These include WGCNA and MEGENA implementation for comparison with MSCN, simulation-based module-recovery benchmarking, down-sampling and covariate-imbalance sensitivity analyses, and processing of an external ASD brain RNA-seq dataset for evaluating externally supported candidate TF-lncRNA-mRNA mediation axes.

[2] Supplementary Tables (Page S4)
This section describes Supplementary Tables S1-S5, organized according to the MSCN framework. These tables provide cohort metadata, multilevel gene co-expression module assignments, results from functional enrichment analyses (GO, KEGG, and SFARI), lncRNA-to-module mappings, transcription factor (TF) enrichment results, candidate TF-lncRNA-mRNA mediation axes, external support for mediation axes, and annotations of named lncRNA mediators.

[3] Supplementary Figures S1-S13 (Pages S5-S17)
This section presents visual materials referenced in the main text, including MSCN algorithm illustrations, hierarchical module structures, cohort-specific DE-module visualizations, covariate correlation and module-covariate association heatmaps, cross-method comparisons with WGCNA and MEGENA, module preservation and enrichment benchmarks, runtime analyses, sample-size and covariate-imbalance sensitivity analyses, MSCN scalability evaluation, inter-edge correlation distributions, and simulation-based MSCN/WGCNA comparisons.

[1] Supplementary Methods

Module detection using established co-expression frameworks: WGCNA and MEGENA

To compare MSCN with established co-expression network frameworks, WGCNA and MEGENA were applied to the same covariate-adjusted mRNA expression matrix in Cohort 1 used for MSCN. WGCNA analysis was performed using the WGCNA R package (version 1.74). For this dataset, the soft-thresholding power was selected using pickSoftThreshold as the first power reaching a scale-free topology fit index $R^2 > 0.85$. Co-expression modules were then detected using blockwiseModules with a minimum module size of 30 and a merge cut height of 0.25; all other parameters were kept at default values. Genes assigned to the WGCNA grey module were considered unassigned and excluded from downstream module-overlap analyses. MEGENA analysis was performed using the MEGENA R package (version 1.3.7). A planar filtered network was first constructed from the input expression matrix, followed by multiscale module detection. The maximum module size was restricted to half of the total number of input genes. Module and hub significance thresholds were set to $P < 0.05$, and all remaining parameters were kept at default values.

Generation of simulated datasets and performance benchmarking

To evaluate module recovery performance under a known ground-truth structure, we generated 50 independent simulated gene expression datasets using the simulateDatExpr function from the WGCNA R package (version 1.74). To make the simulated datasets reflect realistic co-expression structures, empirical module proportions and eigengenes were derived from the primary cohort expression data. WGCNA clustering was performed using the same settings as described above for module detection. Each simulated dataset contained 5,000 genes and was generated using the default simulation parameters of simulateDatExpr. The simulator-provided module labels were used as the ground-truth labels. Clustering performance was quantified by adjusted Rand index (ARI) and normalized mutual information (NMI), calculated using the R package aricode (version 1.0.3). These metrics measured concordance between algorithm-derived module assignments and the simulator-provided ground-truth labels. Between-method differences across the 50 simulation replicates were assessed using a two-sided paired Wilcoxon signed-rank test.

Down-sampling and covariate-imbalance sensitivity analyses

To assess the data conditions under which MSCN-based module-trait inference remains robust, we performed sensitivity analyses using Cohort 1. First, repeated down-sampling was conducted across a range of sample sizes of $n = 100$, 80, and 60. For each down-sampled dataset, samples were selected without replacement while preserving diagnosis balance as closely as possible. The same MSCN module detection and module-diagnosis association pipeline used in the main analysis was then applied. The number of ASD-associated modules passing the same FDR threshold as the main analysis was recorded for each replicate. Second, to evaluate the effect of covariate imbalance, we generated subsampled Cohort 1 datasets with progressively imbalanced distributions of major covariates, including sequencing batch and brain region, at $n = 60$. For each imbalance setting, the proportion of samples from a specified covariate category was varied from approximately balanced to highly imbalanced. MSCN module detection and module-diagnosis association testing were repeated under each setting, and the number of FDR-significant ASD-associated modules was recorded. These analyses were used to distinguish the effects of sample size from those of diagnosis-covariate collinearity and tissue/source heterogeneity on FDR-controlled module-trait discovery.

External ASD brain RNA-seq dataset processing and mediation analysis

For external transcriptomic support analyses, we incorporated an independent ASD brain RNA-seq dataset from Arpi and Simpson (2022). This dataset includes cortical samples spanning multiple brain regions from the Autism Tissue Program brain bank, including BA7, BA17, BA4/6, and BA38. Analyses were restricted to genes or transcripts overlapping with those evaluated in the primary cohorts. To ensure consistency with the discovery analysis, expression values were normalized and corrected using the same covariate-adjustment framework. Technical and biological covariates, including age, sex, RIN, PMI, and brain region, were regressed out using a linear mixed-effects model, with subject identity included as a random effect when repeated regional samples were available. The resulting adjusted expression profiles were used for external evaluation of ASD-associated gene sets and candidate mediation axes. To evaluate the reproducibility of candidate TF-lncRNA-mRNA mediation axes, we repeated the mediation analysis in this external transcriptomic dataset. For each externally testable axis, all three components—the TF, lncRNA, and target mRNA—were required to be detectable in the external dataset. The same mediation criteria used in the discovery analysis were applied to the external dataset. A TF-lncRNA-mRNA axis was considered externally supported if it showed a significant average causal mediation effect (ACME; $P < 0.05$), a significant total effect ($P < 0.05$), a non-significant average direct effect (ADE; $P \geq 0.05$), and concordant directions between the ACME and total effect estimates. Externally supported axes were summarized in Supplementary Table S5b.

[2] Supplementary Tables

Table S1. Sample metadata and covariate-adjusted expression for ASD brain transcriptomic cohorts. This table provides key data inputs for the MSCN analysis, including: (a) Sample metadata for Cohort 1; (b) Sample metadata for Cohort 2; (c) Covariate-adjusted mRNA expression matrix for Cohort 1; (d) Covariate-adjusted mRNA expression matrix for Cohort 2; and (e) Covariate-adjusted lncRNA expression matrix for Cohort 1. Expression values were corrected using a linear mixed-effects model to remove variation attributable to technical and biological confounders unrelated to ASD diagnosis (see Methods for details).

Table S2. Multilevel MSCN module assignments and network structures in ASD transcriptomic cohorts. This table presents the hierarchical gene co-expression modules identified using the MSCN algorithm across four levels of resolution. (a) Gene-to-module assignments for Cohort 1, listing each gene with its corresponding module membership across MSCN Levels 1-4; (b) Gene-to-module assignments for Cohort 2, listing each gene with its corresponding module membership across MSCN Levels 1-4; (c) Inferred gene−gene co-expression edge list for Cohort 1, showing all pairwise edges retained in the MSCN framework at each hierarchical level, including edge weights and level identifiers; and (d) Inferred gene−gene co-expression edge list for Cohort 2, in the same format as (c). Module construction was performed independently in each cohort. Edge distances were computed from covariate-adjusted expression matrices and used to merge gene clusters into modules recursively.

Table S3. ASD-associated DE-modules and their functional enrichment results. This table includes the MSCN-derived gene co-expression modules significantly associated with ASD diagnosis in each cohort, based on eigengene-level differential expression analyses. (a) DE-modules from Cohort 1 include module identifiers, statistical significance, enriched GO terms, KEGG pathways (with enrichment *P* values), and hypergeometric *P* values for SFARI gene enrichment. (b) ASD-associated modules from Cohort 2 are in the same format as (a).

Table S4. Mapping of lncRNAs to MSCN-derived mRNA co-expression modules in ASD. This table reports the mapping of lncRNAs into the modular architecture inferred by MSCN in Cohort 1, providing a basis for downstream TF and mediation analyses. Each lncRNA was assigned to a module based on its nearest-neighbor mRNA(s), defined by the highest expression correlation, thereby linking noncoding transcripts to disease-relevant networks.

Table S5. Candidate transcription factor (TF)-lncRNA-mRNA mediation axes identified by mediation analysis within ASD-associated modules. This table reports a multiscale mediation landscape emerging from ASD-associated MSCN modules, integrating TF enrichment, lncRNA assignment (from Table S4), and statistical mediation analysis to infer putative TF-lncRNA-mRNA axes. (a) TF enrichment for ASD modules (identified in Table S3). (b) Significant TF-lncRNA-mRNA mediation axes. (c) Annotation of 12 named lncRNA mediators identified in SFARI-related TF-lncRNA-mRNA mediation axes.

[3] Supplementary Figures

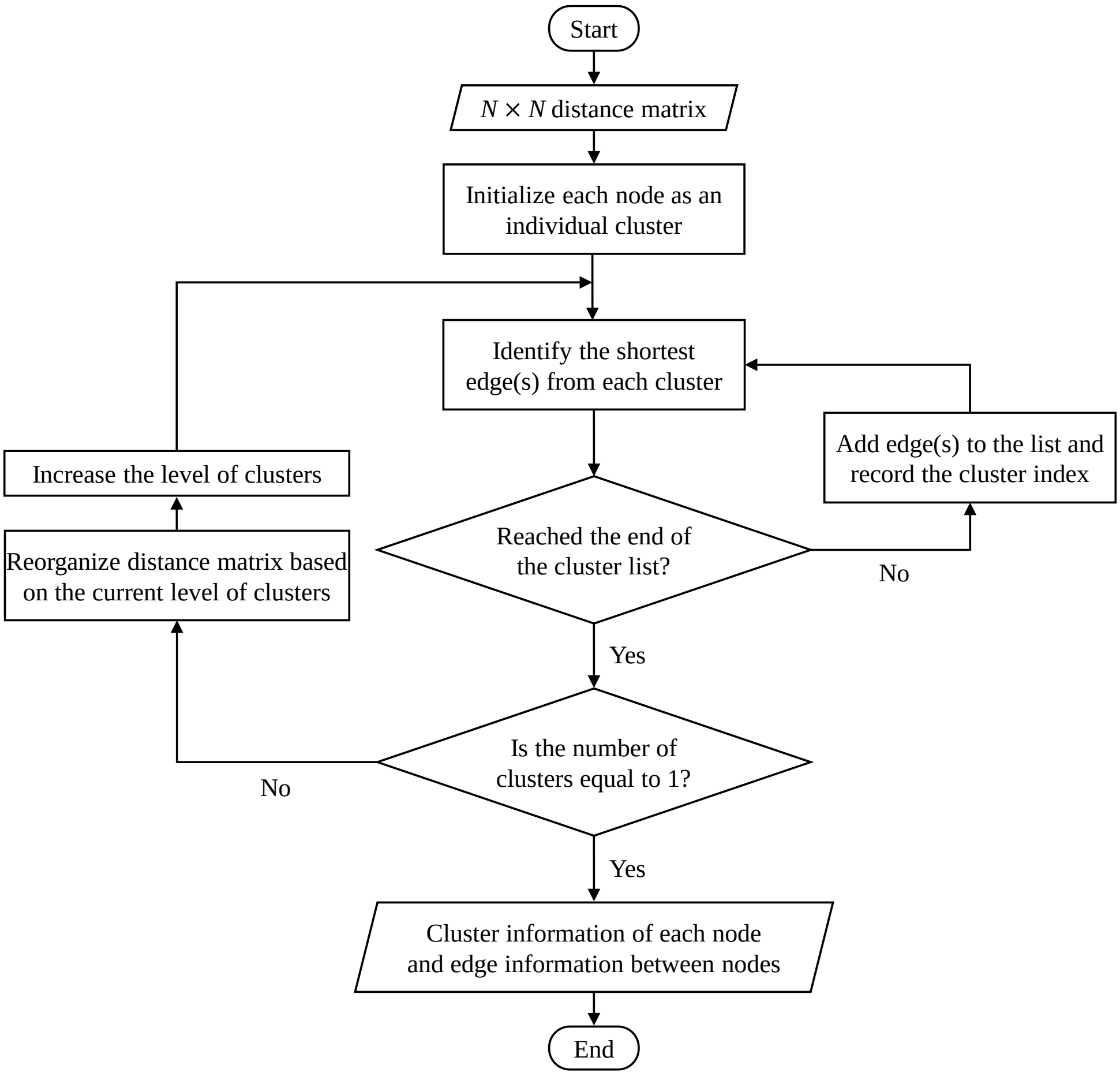

Supplementary Figure S1. Flowchart of the multilevel MSCN algorithm. This schematic outlines the iterative procedure of the Minimum Span Clustering Network (MSCN) algorithm used for multilevel gene co-expression module identification.

a

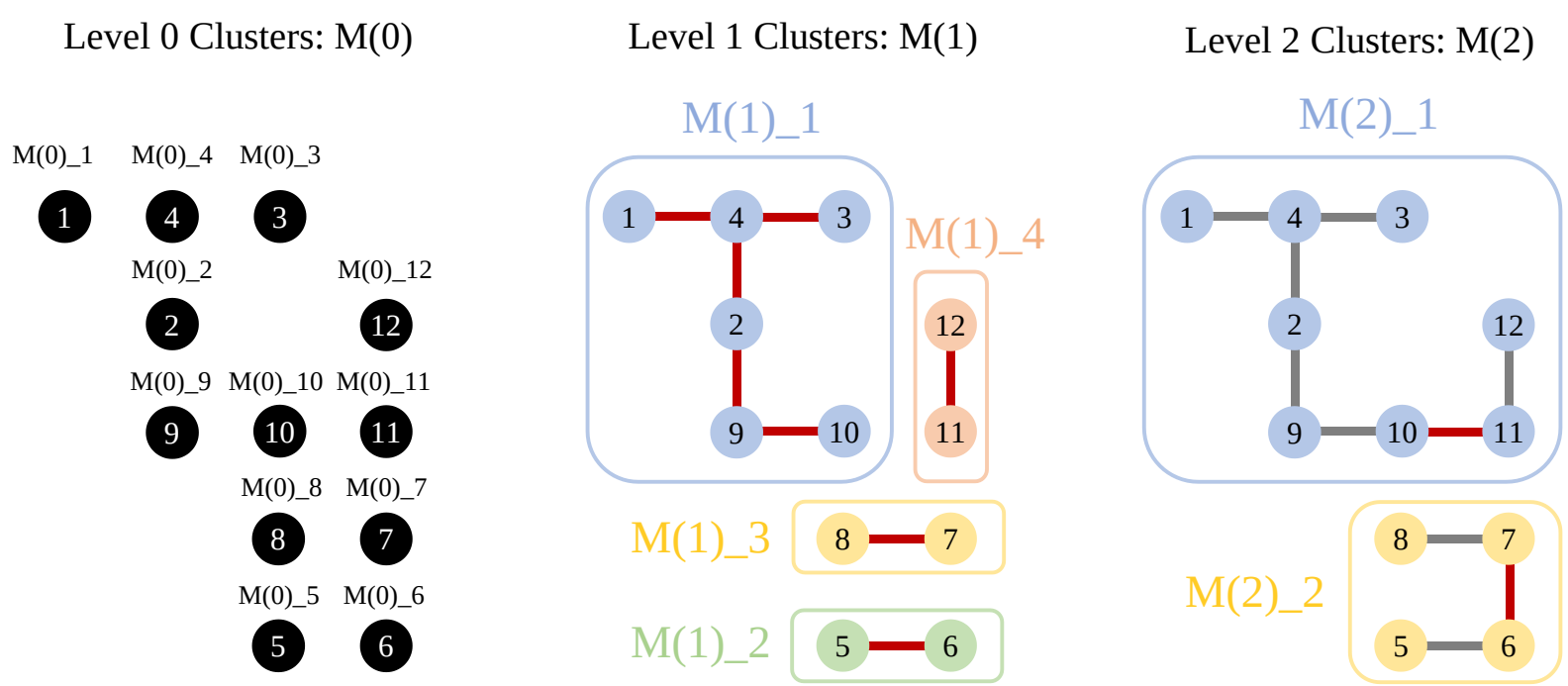

b

Node1	Node2	Displayed Distance	Level
M(0)_1	M(0)_4	14	1
M(0)_2	M(0)_4	6	1
M(0)_3	M(0)_4	2	1
M(0)_5	M(0)_6	22	1
M(0)_7	M(0)_8	18	1
M(0)_2	M(0)_9	8	1
M(0)_9	M(0)_10	14	1
M(0)_11	M(0)_12	10	1
M(0)_10	M(0)_11	22	2
M(0)_6	M(0)_7	26	2

c

Node	Level:1	Level:2
M(0)_1	M(1)_1	M(2)_1
M(0)_2	M(1)_1	M(2)_1
M(0)_3	M(1)_1	M(2)_1
M(0)_4	M(1)_1	M(2)_1
M(0)_5	M(1)_2	M(2)_2
M(0)_6	M(1)_2	M(2)_2
M(0)_7	M(1)_3	M(2)_2
M(0)_8	M(1)_3	M(2)_2
M(0)_9	M(1)_1	M(2)_1
M(0)_10	M(1)_1	M(2)_1
M(0)_11	M(1)_4	M(2)_1
M(0)_12	M(1)_4	M(2)_1

Supplementary Figure S2. Schematic illustration of the hierarchical clustering process in the MSCN framework. (a) This toy example corresponds to the overall workflow shown in main-text Figure 1, conceptualizing how MSCN constructs multiscale modular structures from gene expression data. Each node is initialized as an individual cluster at Level 0 (M(0)). In Level 1 (M(1)), clusters are iteratively merged based on the shortest inter-node distances (red edges), forming intermediate modules (e.g., M(1)_1 to M(1)_4). These Level 1 modules are further merged into Level 2 modules (M(2)) based on their minimal distances. Nodes belonging to each module are highlighted with colored outlines to indicate their hierarchical membership. (b) The corresponding edge list used for cluster merging at each level shows node pairs, displayed edge distances, and the hierarchical level at which merging occurred. (c) A cluster membership table maps each original node (M(0)) to its assigned module at Levels 1 and 2. The edge distances shown in (b) are display-scaled toy distances, $100 \times d_{ij}$, where $d_{ij} = 1 - | r_{ij} |$. The integer values are not unscaled empirical distances but are used to simplify visualization of the hierarchical merging process.

a

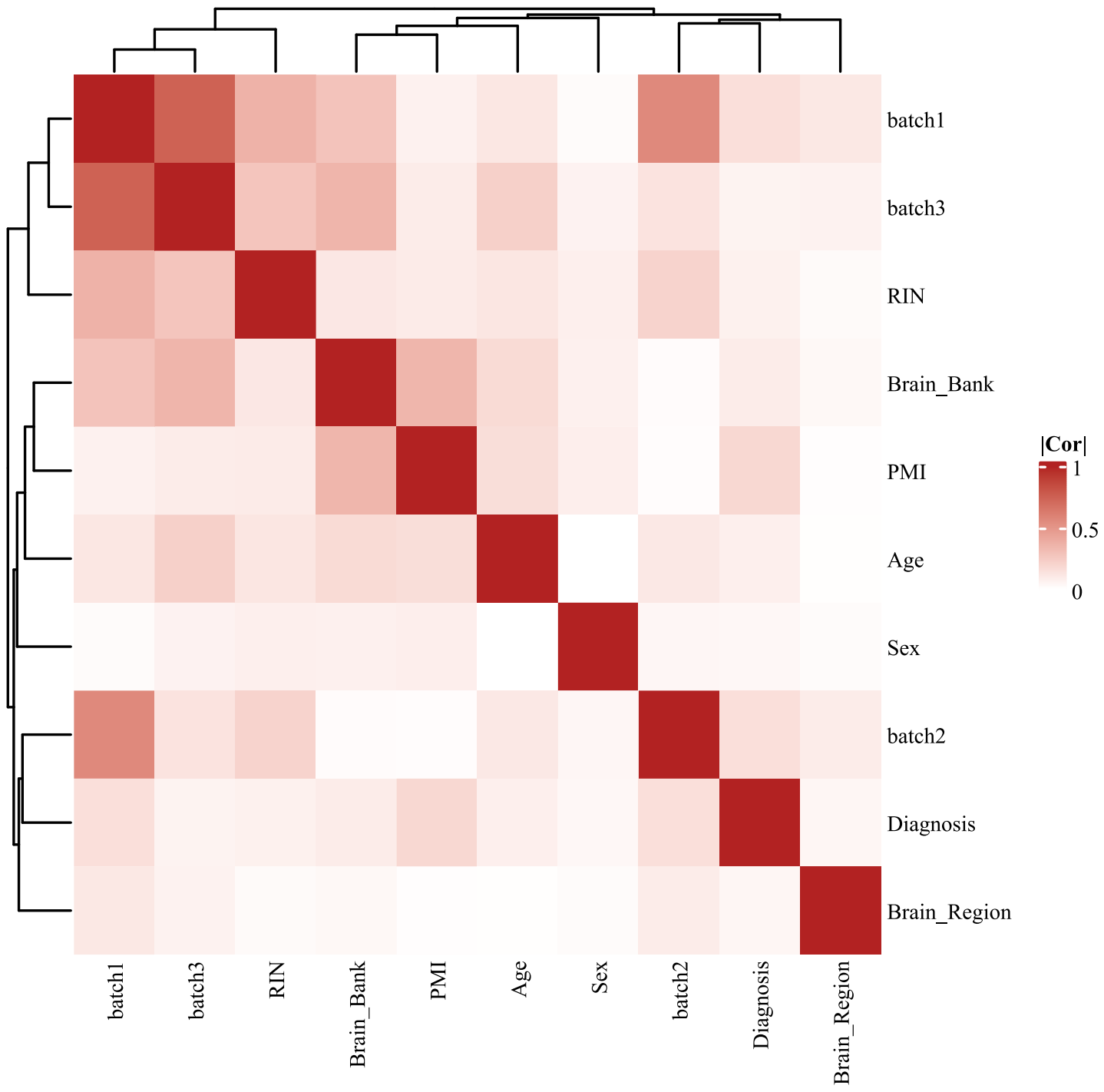

b

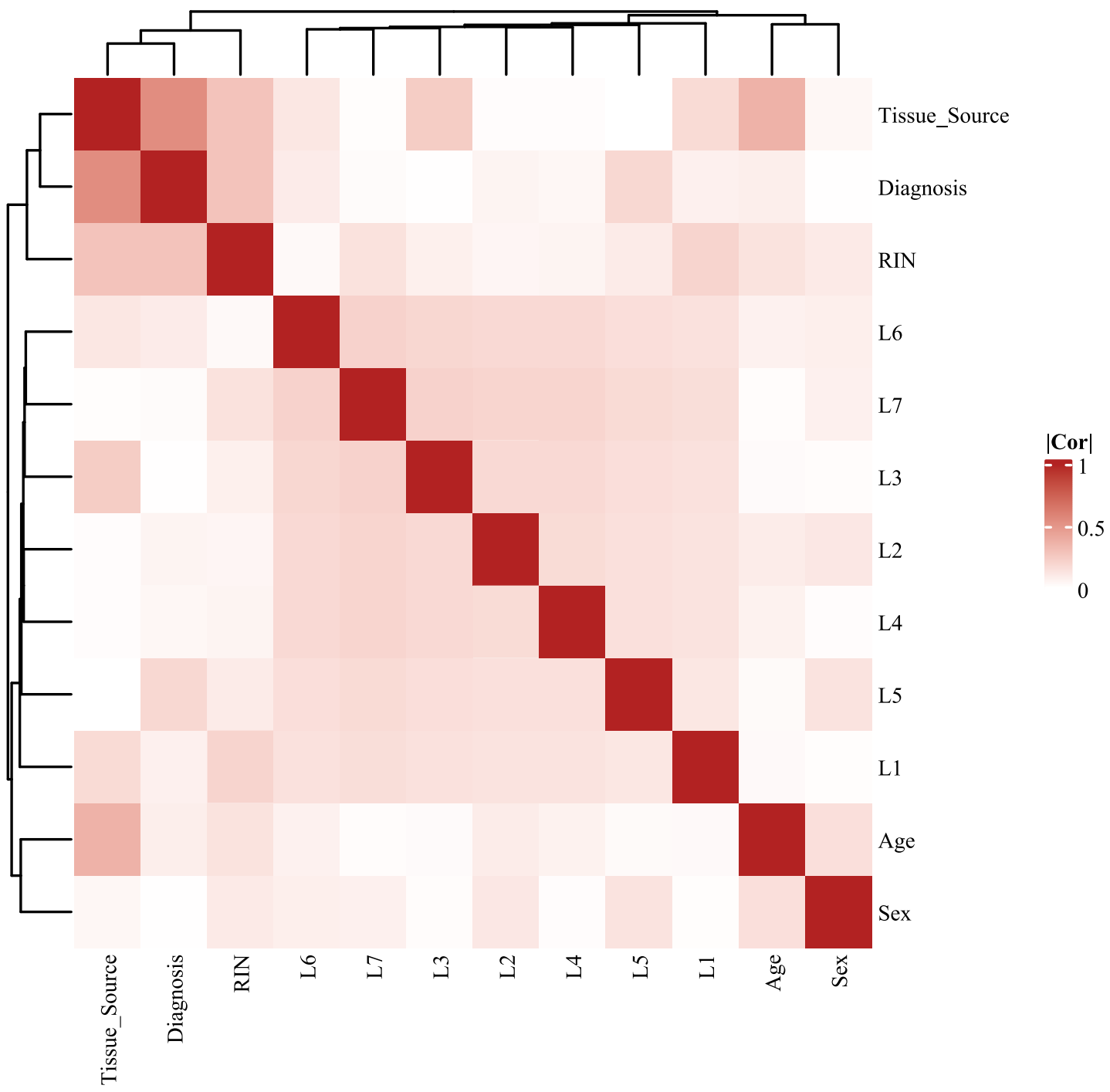

Supplementary Figure S3. Pairwise correlation matrix of sample covariates across cohorts. Pairwise correlation heatmap of technical and biological covariates in Cohort 1 (a) and Cohort 2 (b). Covariates were encoded using one-hot encoding for categorical variables before computing pairwise Pearson correlation coefficients. Hierarchical clustering was applied to both axes to group interrelated covariates.

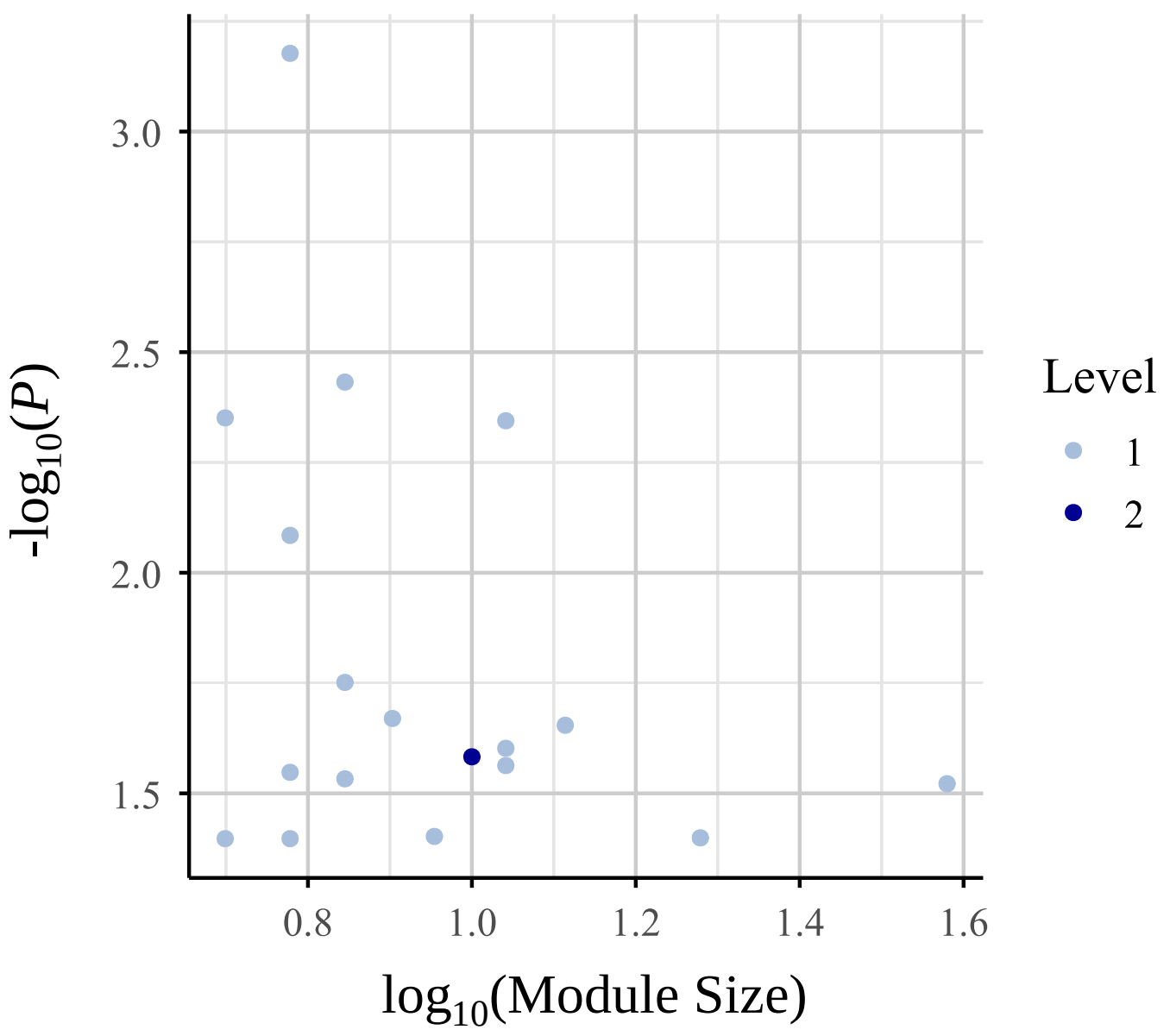

Supplementary Figure S4. Relationship between module size and ASD-association **significance across MSCN levels.** Scatter plot showing the relationship between module size and ASD-association significance among ASD-associated modules in Cohort 2. Each point denotes a module, and color indicates the MSCN hierarchy level (light to dark blue for Levels 1 to 2).

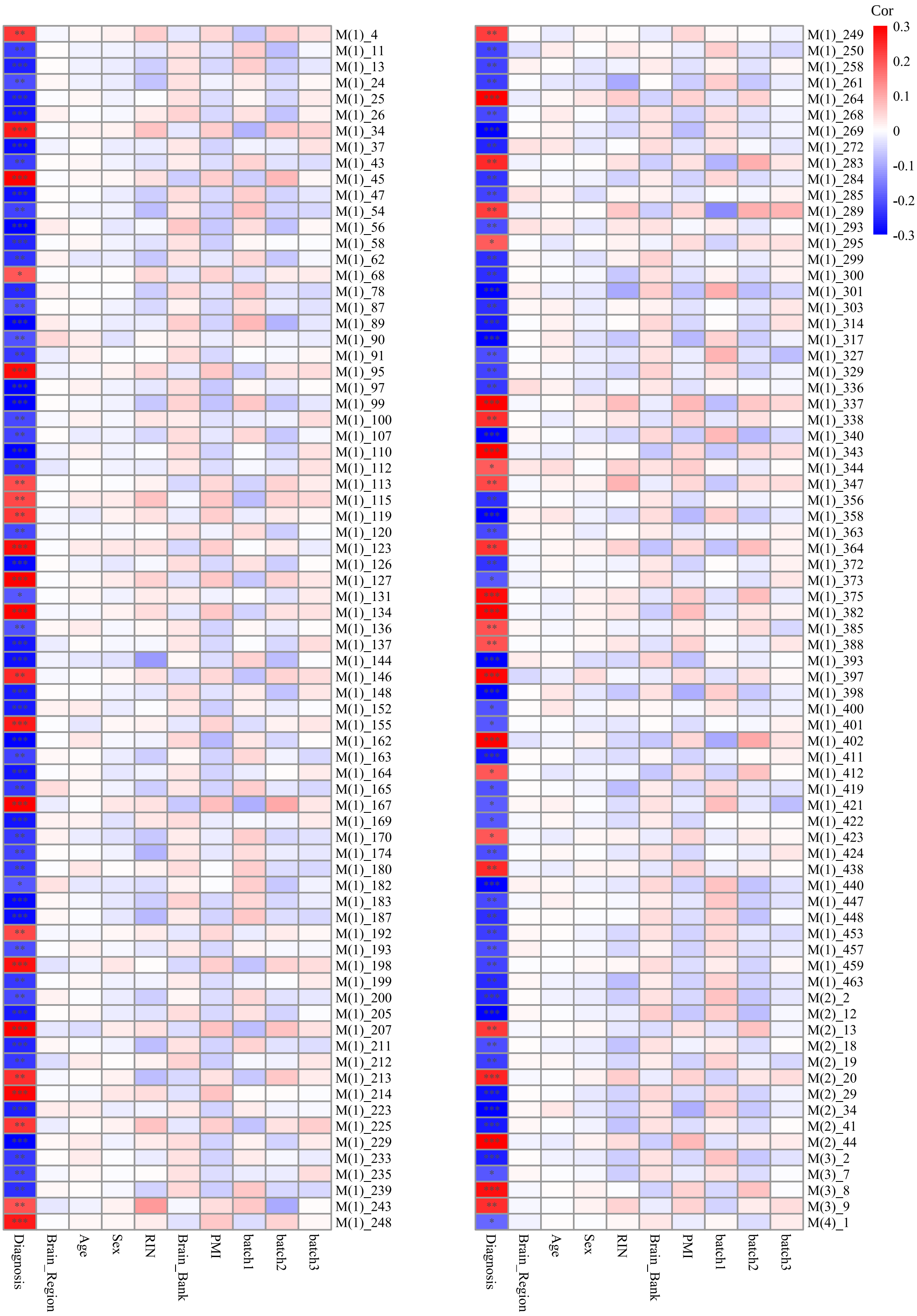

Supplementary Figure S5. Correlation between module eigengenes and covariates in ASD (Cohort 1). Heatmap showing Pearson correlation coefficients between module eigengenes (rows) and sample-level covariates (columns) for ASD-associated modules at MSCN Levels 1 to 4 in Cohort 1. The color scale reflects the correlation direction and magnitude. Statistical significance is indicated: $P < 0.05$ (*), $P < 0.01$ (**), $P < 0.001$ (***).

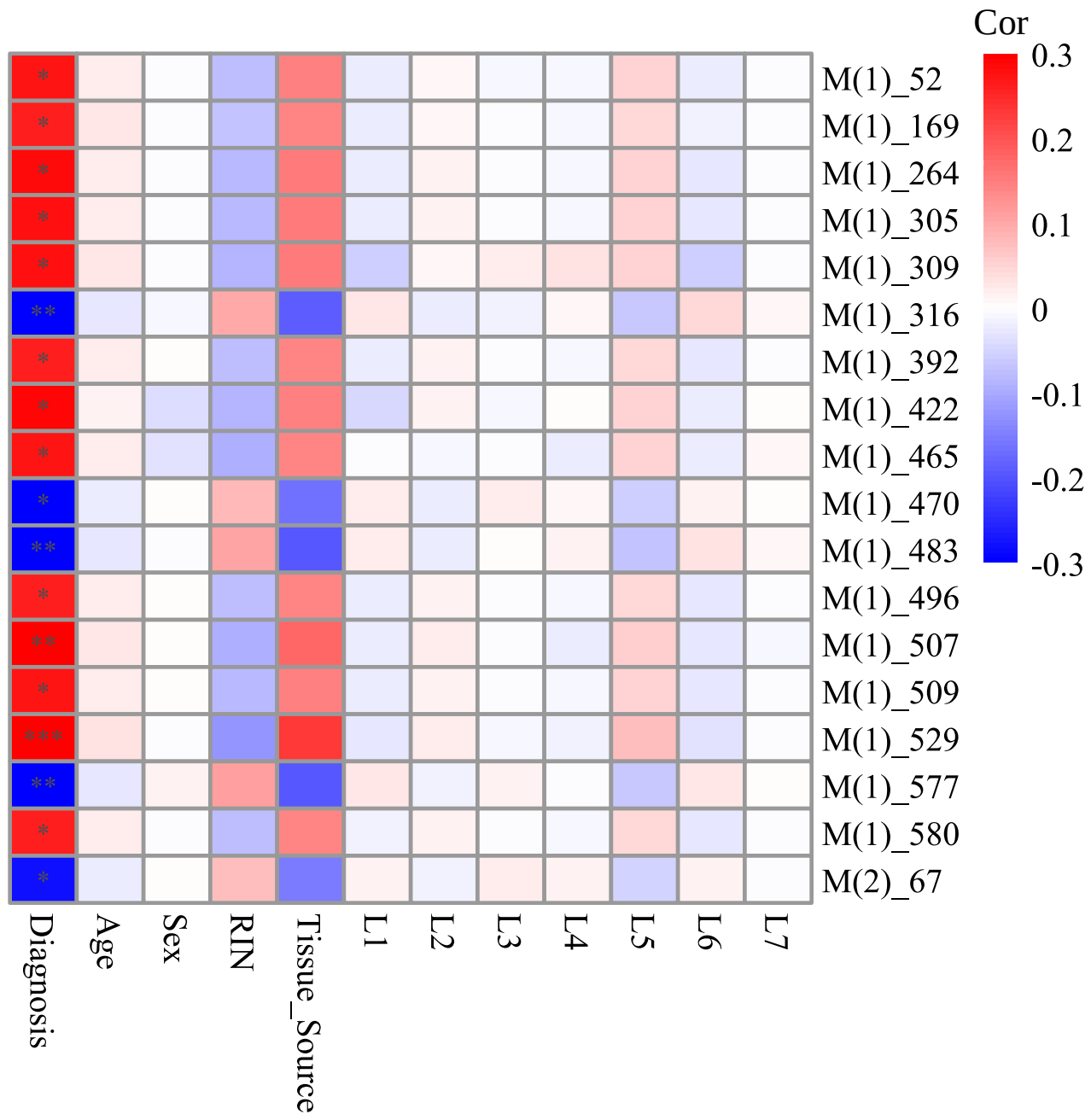

Supplementary Figure S6. Correlation between module eigengenes and covariates in ASD (Cohort 2). Heatmap showing Pearson correlation coefficients between module eigengenes (rows) and sample-level covariates (columns) for ASD-associated modules at MSCN Levels 1 to 2 in Cohort 2. The color scale reflects the correlation direction and magnitude. Statistical significance is indicated: $P < 0.05$ (*), $P < 0.01$ (**), $P < 0.001$ (***).

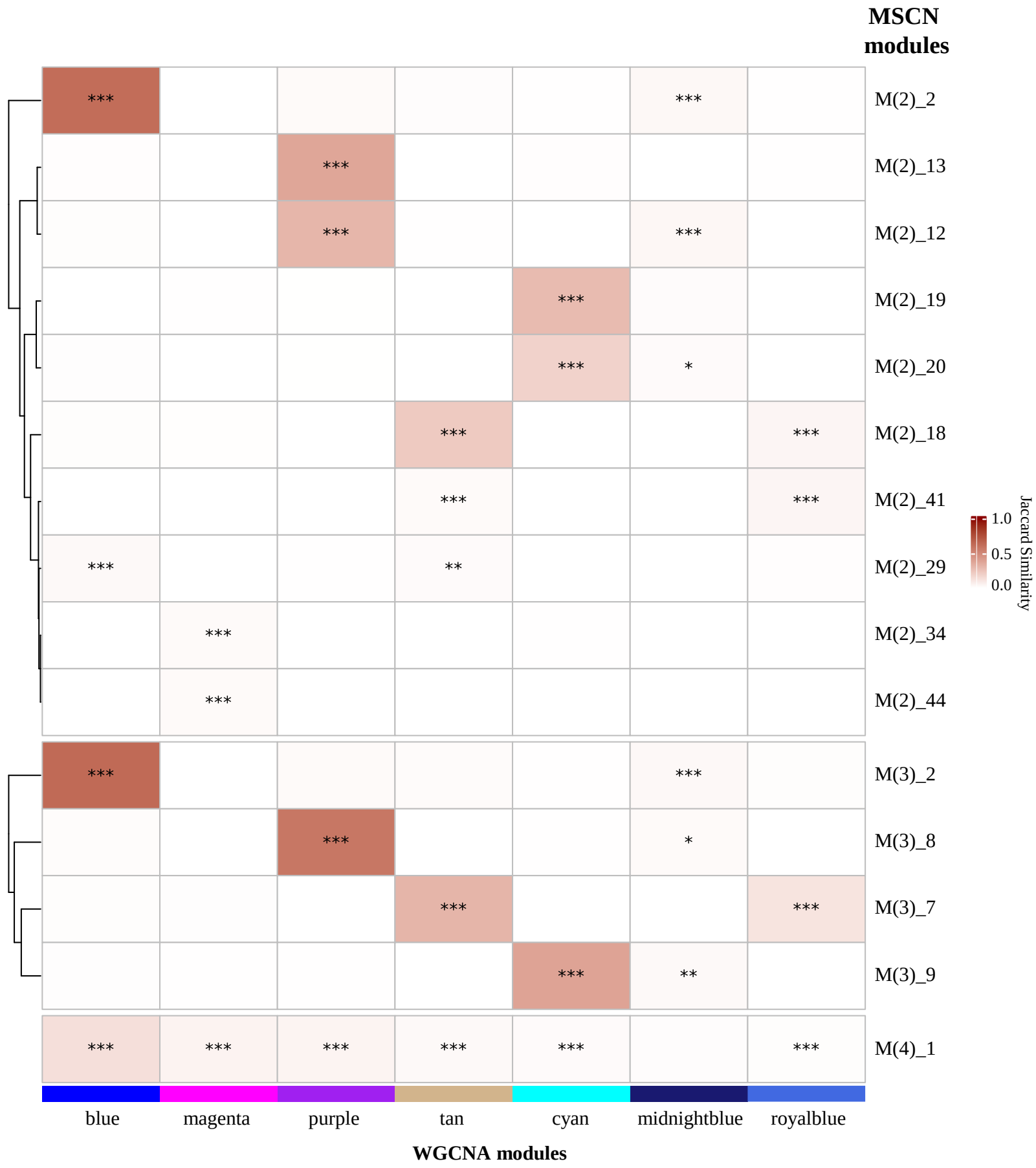

Supplementary Figure S7. Concordance between Cohort 1 MSCN ASD-associated modules and WGCNA modules significantly associated with ASD. Heatmap showing Jaccard similarities between Cohort 1 MSCN ASD-associated DE-modules at Levels 2-4 and ASD-significant WGCNA modules identified from the same covariate-adjusted mRNA expression matrix. Rows represent MSCN modules and columns represent WGCNA color-labeled modules. Statistical significance of module overlap was assessed by hypergeometric testing. Asterisks indicate significance levels: *$P < 0.05$, **$P < 0.01$, and ***$P < 0.001$.

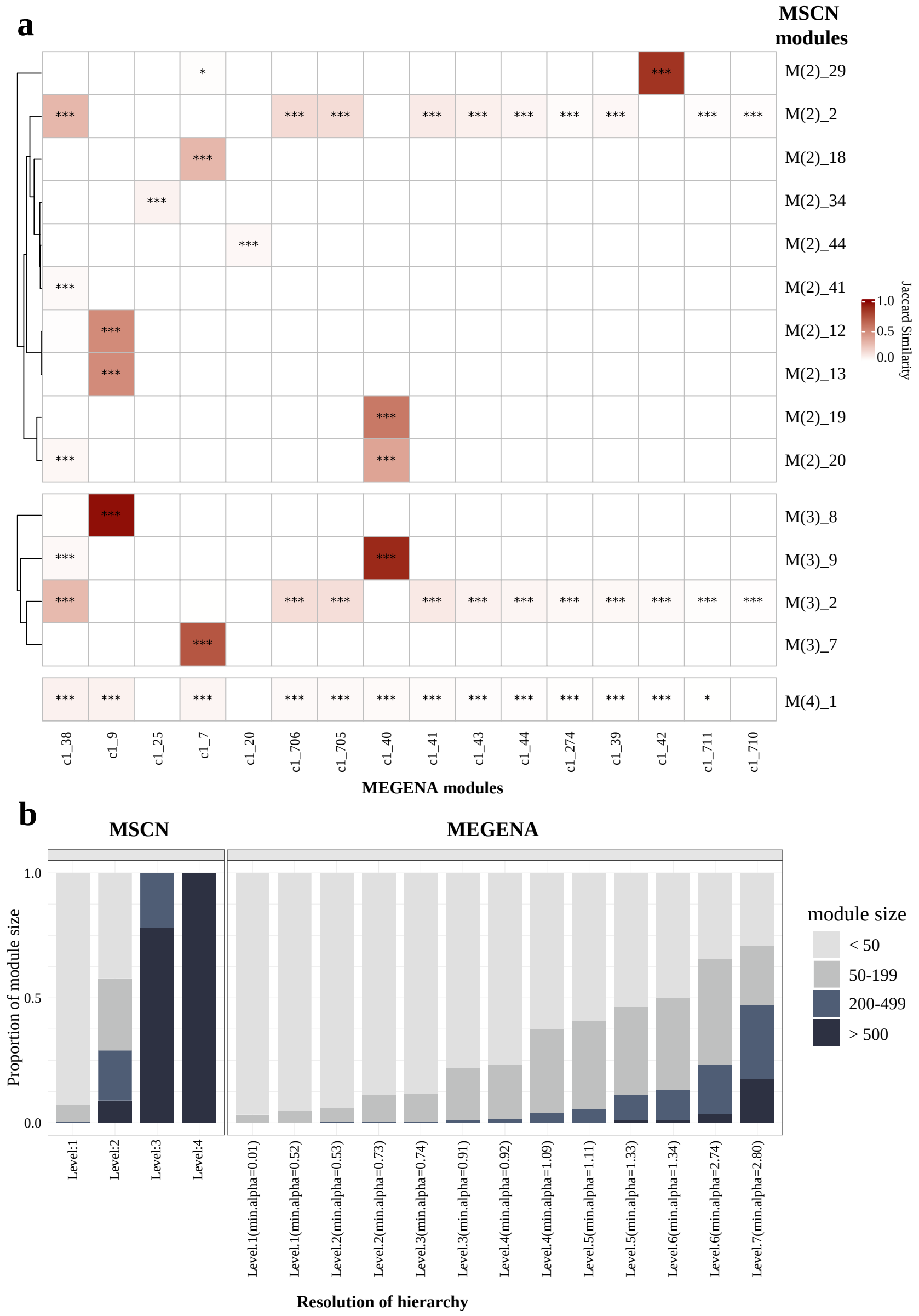

Supplementary Figure S8. Comparison between MSCN and MEGENA representations of ASD-associated co-expression modules. (a) Heatmap showing Jaccard similarities between Cohort 1 MSCN ASD-associated DE-modules at Levels 2-4 and MEGENA modules constructed from the same covariate-adjusted mRNA expression matrix. Rows represent MSCN modules and columns represent MEGENA modules. Statistical significance of module overlap was assessed by hypergeometric testing. Asterisks denote *$P < 0.05$, **$P < 0.01$, and ***$P < 0.001$. (b) Distribution of module-size categories across MSCN and MEGENA hierarchical resolutions. Stacked bars show the proportion of modules in each size category: < 50, 50-199, 200-499, and > 500 genes.

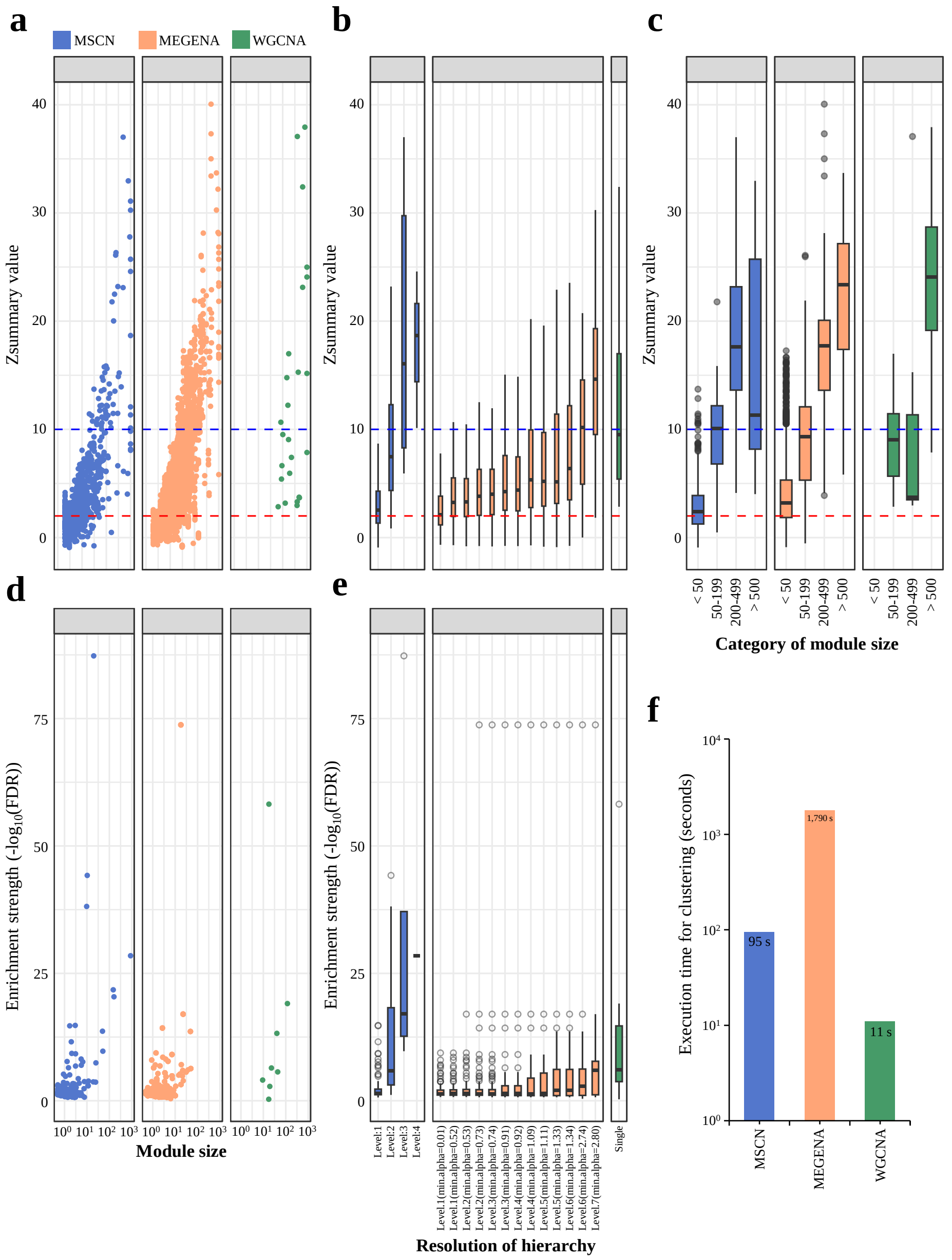

Supplementary Figure S9. Benchmark comparison of MSCN, MEGENA, and WGCNA in terms of module preservation, enrichment strength, and runtime in Cohort 1. (a) Scatter plots showing module preservation Zsummary values as a function of module size for MSCN, MEGENA, and WGCNA, using the modulePreservation function from WGCNA. Dashed reference lines indicate commonly used preservation thresholds, with Zsummary > 10 representing strong preservation and Zsummary > 2 representing weak-to-moderate preservation. (b) Distribution of Zsummary values across hierarchical resolutions for each method. (c) Distribution of Zsummary values stratified by module-size category: < 50, 50-199, 200-499, and > 500 genes. (d) Scatter plots showing enrichment strength, defined as $-\log_{10}(\mathrm{FDR})$, as a function of module size for each method. (e) Distribution of enrichment strength across hierarchical resolutions. (f) Runtime comparison for module construction only.

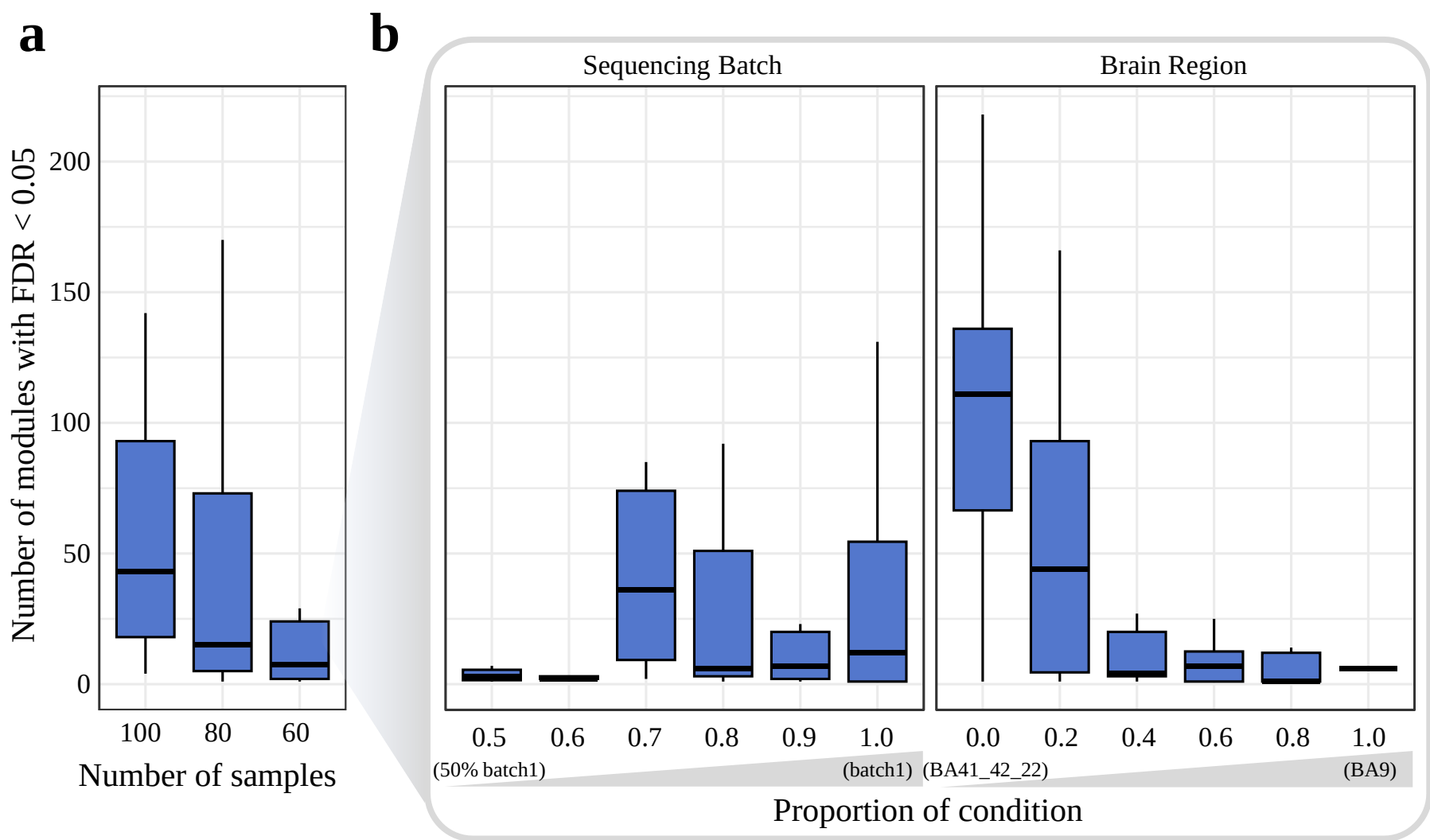

Supplementary Figure S10. Sensitivity analysis of MSCN-based ASD module discovery under reduced sample sizes and covariate imbalance. (a) Repeated down-sampling analysis of Cohort 1. Boxplots show the number of FDR-significant ASD-associated modules detected after reducing the sample size to $n = 100$, 80, or 60. (b) Covariate-imbalance analysis evaluating the effect of sequencing-batch and brain-region composition on FDR-controlled ASD module discovery at $n = 60$. Boxplots show the number of modules with FDR < 0.05 across repeated subsampling under different covariate compositions. For sequencing batch, 0.5 indicates balanced representation and 1.0 indicates samples drawn entirely from batch 1. For brain region, 0.0 indicates samples drawn entirely from BA41/42/22 and 1.0 indicates samples drawn entirely from BA9.

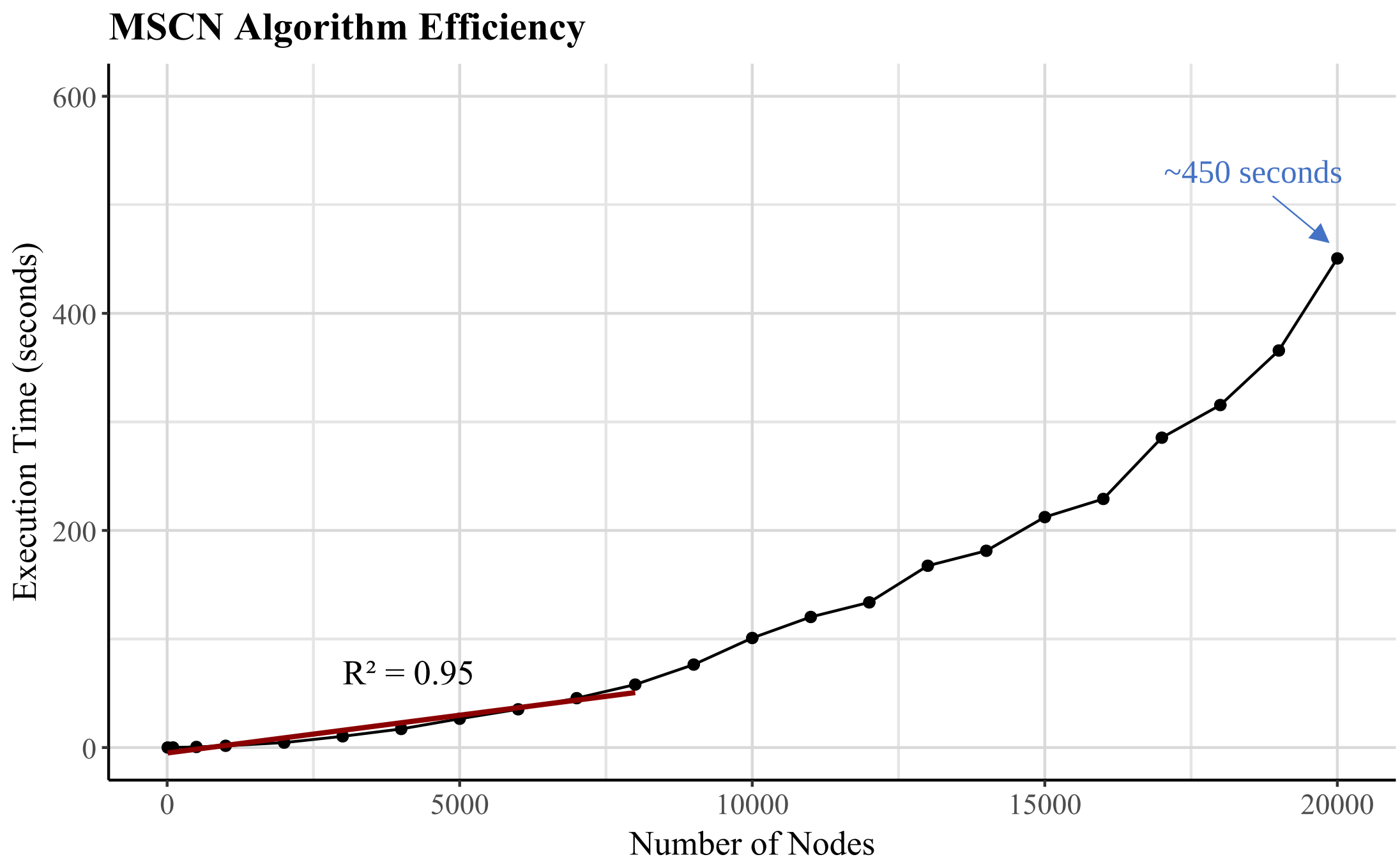

Supplementary Figure S11. Computational efficiency of the MSCN algorithm. Execution time (in seconds) required to run MSCN as a function of the number of input nodes (genes). The algorithm showed approximately linear scaling up to ~8,000 genes ($R^2 = 0.95$) and remained computationally feasible at transcriptome scale, requiring approximately 450 seconds for 20,000 genes.

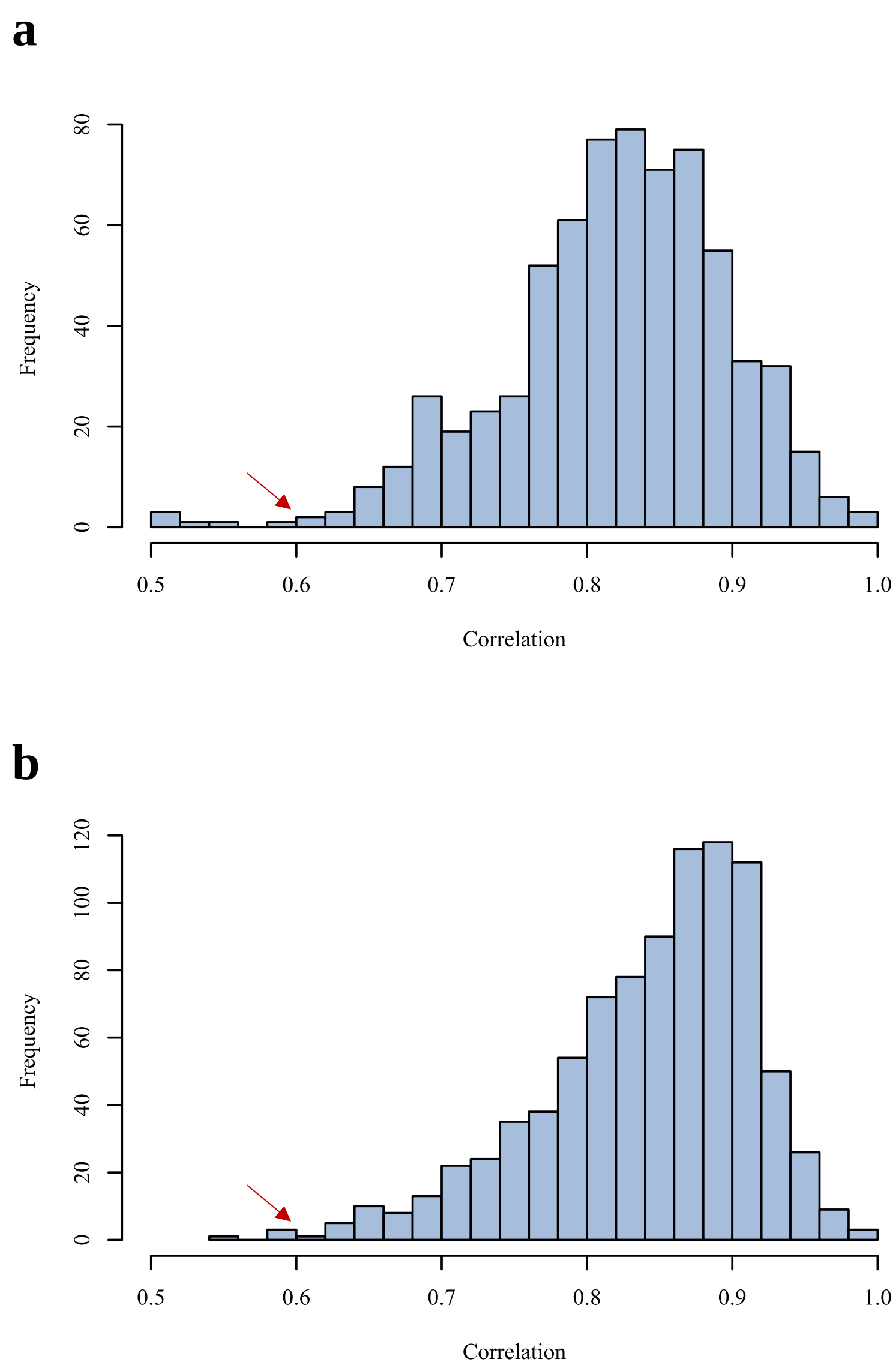

Supplementary Figure S12. **Distribution of inter-cluster edge correlations across hierarchical MSCN modules.** Histogram of Pearson correlation coefficients for inter-module gene-gene edges in MSCN at Levels 2 and above, derived from (a) Cohort 1 and (b) Cohort 2. The distributions reveal a sharp decline in correlation values near $|r| = 0.6$.

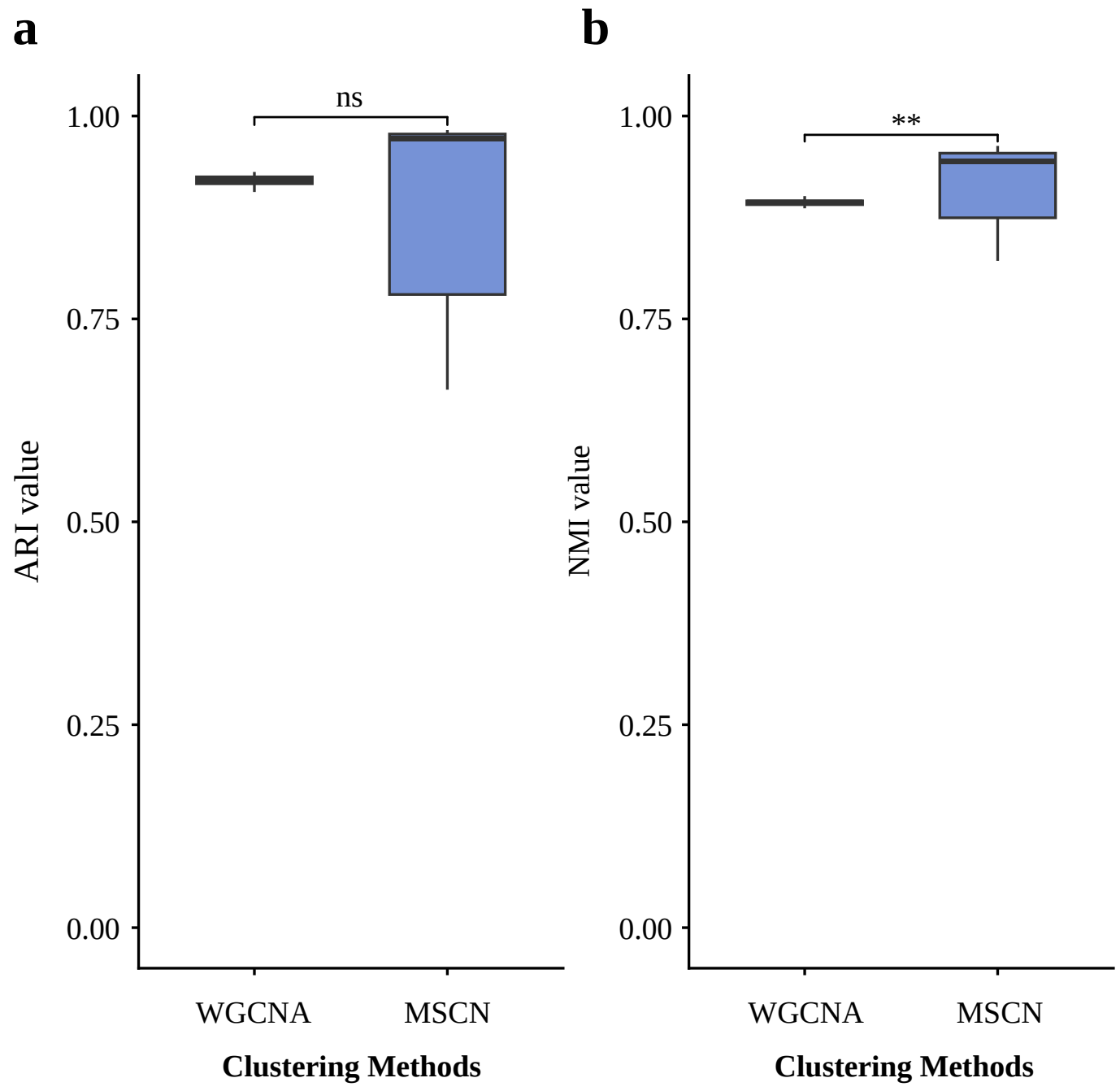

Supplementary Figure S13. Simulation-based comparison of MSCN and WGCNA in flat-module data generated by WGCNA. Simulated expression matrices with known module labels were generated using the built-in simulation utility of the WGCNA package. MSCN and WGCNA were applied to the same simulated datasets, and clustering accuracy was evaluated against the simulator-provided ground-truth labels using (a) adjusted Rand index (ARI) and (b) normalized mutual information (NMI). Each boxplot summarizes results across 50 independent simulation replicates. Statistical significance was assessed using a two-sided paired Wilcoxon signed-rank test. ns, not significant; $^{**}P < 0.01$.
